# ANIMAL-SPOT enables animal-independent signal detection and classification using deep learning

**DOI:** 10.1038/s41598-022-26429-y

**Published:** 2022-12-19

**Authors:** Christian Bergler, Simeon Q. Smeele, Stephen A. Tyndel, Alexander Barnhill, Sara T. Ortiz, Ammie K. Kalan, Rachael Xi Cheng, Signe Brinkløv, Anna N. Osiecka, Jakob Tougaard, Freja Jakobsen, Magnus Wahlberg, Elmar Nöth, Andreas Maier, Barbara C. Klump

**Affiliations:** 1 Pattern Recognition Lab, Department of Computer Science, Friedrich-Alexander-Universität Erlangen-Nürnberg, 91058 Erlangen, Germany; 2 Cognitive and Cultural Ecology Lab, Max Planck Institute of Animal Behavior, 78315 Radolfzell, Germany; 3 Department of Human Behavior, Ecology and Culture, Max Planck Institute for Evolutionary Anthropology, 04103 Leipzig, Germany; 4 Biology Department, University of Konstanz, 78464 Constance, Germany; 5 Department of Natural Resources and Environmental Sciences, University of Illinois Urbana-Champaign, Champaign, IL United States; 6 Max Planck Institute for Biological Intelligence, in Foundation, Seewiesen Eberhard-Gwinner-Strasse, 82319 Starnberg, Germany; 7 Department of Anthropology, University of Victoria, Victoria, BC V8P 5C2 Canada; 8 Leibniz Institute for Zoo and Wildlife Research, Alfred-Kowalke-Straße 17, 10315 Berlin, Germany; 9 Department of Bioscience, Wildlife Ecology, Aarhus University, 8410 Rønde, Denmark; 10 Department of Vertebrate Ecology and Zoology, Faculty of Biology, University of Gdańsk, 80-308 Gdańsk, Poland; 11 Department of Bioscience, Marine Mammal Research, Aarhus University, 4000 Roskilde, Denmark; 12 Department of Biology, University of Southern Denmark, 5230 Odense, Denmark

**Keywords:** Classification and taxonomy, Machine learning, Software

## Abstract

Bioacoustic research spans a wide range of biological questions and applications, relying on identification of target species or smaller acoustic units, such as distinct call types. However, manually identifying the signal of interest is time-intensive, error-prone, and becomes unfeasible with large data volumes. Therefore, machine-driven algorithms are increasingly applied to various bioacoustic signal identification challenges. Nevertheless, biologists still have major difficulties trying to transfer existing animal- and/or scenario-related machine learning approaches to their specific animal datasets and scientific questions. This study presents an animal-independent, open-source deep learning framework, along with a detailed user guide. Three signal identification tasks, commonly encountered in bioacoustics research, were investigated: (1) target signal vs. background noise detection, (2) species classification, and (3) call type categorization. ANIMAL-SPOT successfully segmented human-annotated target signals in data volumes representing 10 distinct animal species and 1 additional genus, resulting in a mean test accuracy of 97.9%, together with an average area under the ROC curve (AUC) of 95.9%, when predicting on unseen recordings. Moreover, an average segmentation accuracy and F1-score of 95.4% was achieved on the publicly available BirdVox-Full-Night data corpus. In addition, multi-class species and call type classification resulted in 96.6% and 92.7% accuracy on unseen test data, as well as 95.2% and 88.4% regarding previous animal-specific machine-based detection excerpts. Furthermore, an Unweighted Average Recall (UAR) of 89.3% outperformed the multi-species classification baseline system of the ComParE 2021 Primate Sub-Challenge. Besides animal independence, ANIMAL-SPOT does not rely on expert knowledge or special computing resources, thereby making deep-learning-based bioacoustic signal identification accessible to a broad audience.

## Introduction

In order to gain deeper insights and a better understanding about animal communication, it is imperative to identify vocalization prototypes, derive linguistic patterns, and correlate acoustic paradigms with corresponding behavioral observations. Therefore, it is mandatory to perform in-depth data analysis of large-scale bioacoustic data archives in order to draw statistically significant and representative hypotheses regarding the vocal repertoire of a particular species. Passive Acoustic Monitoring (PAM) concepts^1–3^ are widely used to acquire massive bioacoustic data collections^4–7^, without affecting the natural animal habitats^8^ and thus significantly increase the probability to observe all natural communicative patterns, following the observer’s paradox principle^9^. Furthermore, PAM-based approaches strongly benefit from decreasing costs for recording equipment and data storage^10–13^, combined with recent technological advances^14–20^. However, time- and human-resource restrictions prohibit a profound and comprehensive manual data analysis. Consequently, machine (deep) learning approaches are increasingly applied in bioacoustic research^21,22^ and have shown to be a productive avenue to identify target animal species (e.g., marine mammals^23–26^, birds^27,28^, bats^29^, mosquitos^30^), smaller acoustic units such as call types (e.g., bird call types^27,31^) and group-level differences within target animal species (e.g., killer whale pods^32^). Despite a growing deployment of various machine (deep) learning techniques in the field of bioacoustics, essential research tasks such as target species identification and call type classification still prove to be extremely difficult and challenging.

Machine (deep) learning approaches are often designed for a particular animal species and lack data-related model adaption and hyperparameter fine-tuning options. In addition, the software and/or source code is often not publicly available, combined with missing or insufficient user guidelines which describe required data preparation, network training setup, and model evaluation. It thereby often precludes not just a general transfer to animal- and user-specific research questions, but mainly prevents non-computer science operators to train their own use-case and animal-specific models, which in turn significantly hampers progress in research on animal communication. In this study, we introduce ANIMAL-SPOT, an open-source machine learning framework that enables biologists to independently train and evaluate animal-specific deep learning-based classification models in order to address fundamental biological research questions, including target/noise detection and/or species/call type identification.

Three typical scenarios present themselves when attempting to identify the vocalizations of a target species or individual: (1) The target signal appears without confounding factors such as other similar vocalizations and the task is to determine the target signal with respect to background noise, (2) the target signal appears in conjunction with other, dissimilar, species-specific vocalizations and the signal of interest must be distinguished between other bioacoustic signals and background noise, and (3) the target signal appears with other signals, some of which share similar properties to the target vocalizations and the model must differentiate between similar signals, dissimilar signals, as well as background noise. The approach described here allows a researcher to address all of these tasks, with slight differences in data structure as well as usage of the trained models.

**Figure 1:**
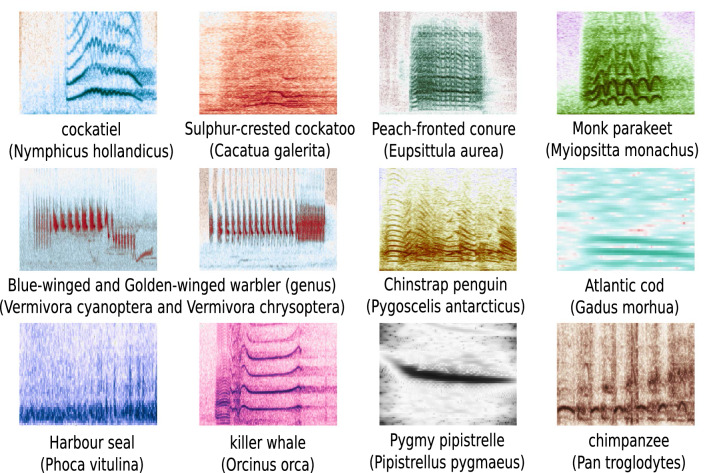
Animal-specific data (10 different species, 1 additional genus) utilized to investigate the ANIMAL-SPOT framework (created via Inkscape^39^, Version 0.92.3).

A detailed user guide^33^, provided in conjunction with this work which describes the data setup as well as model configuration, allows users to create and apply models with no prior deep learning knowledge. The core deep learning workflow took inspiration from ORCA-SPOT^34^, a ResNet-18^35^-based Convolutional Neural Network (CNN), originally designed for segmenting killer whale (*Orcinus orca*) vocalizations from environmental background noise. ANIMAL-SPOT has been adapted and extended to become an animal-independent deep learning framework, evaluating bioacoustic target versus environmental noise detection for 10 species-specific data volumes and 1 additional genus-based dataset, next to the publicly available BirdVox-Full-Night^36^ repository. In addition, multi-species classification has been performed in two different scenarios: (1) as a downstream process, using previously machine-detected and extracted genus-specific target signals, and (2) as a stand-alone procedure, analyzing the Computational Paralinguistics Challenge Primate (ComParE-PRS)^37,38^ multi-species data volume. Moreover, multi-class call type classification has been exemplarily conducted for a single species, using the same downstream approach. The ANIMAL-SPOT workflow is generalizable, enabling unparalleled flexibility in processing task- and animal-specific bioacoustic data corpora. Figure 1 visualizes all animal species-specific spectrograms (10 different species, 1 additional genus), representing a single vocalization event.

In summary, ANIMAL-SPOT provides a publicly-available animal-independent bioacoustic machine learning environment, which allows scientists, regardless of their technical backgrounds, to train and evaluate species-specific deep neural networks, supported by detailed user guidelines, in order to answer fundamental biological research questions (target/sound detection, species classification, and call type recognition). To the best of the authors’ knowledge, this is the first study presenting an animal-independent and publicly-available deep learning framework, evaluated across many bioacoustic signal identification scenarios. As input data, we use many annotated datasets from a broad range of animal species and provide evaluation results using these as well as publicly available species detection and classification challenge data corpora.

## Materials and methods

### Bioacoustic signal identification and classification scenarios

Signal identification can be performed at different levels: (1) taxonomic group (e.g., all birds), (2) species (e.g., monk parakeet), or (3) call type (e.g., contact call). The level highly influences data preparation and classification complexity. Raising taxonomic specialization simultaneously leads to an increase of the labeled data granularity being required for an adequate network training. Furthermore, classification intricacy grows with the level of taxonomic detail. The network has to learn and derive features, being robust against all potential types of environmental noise as well as other animal sounds, except the signals of interest, including within-species variation. The level of taxonomy also affects the amount of chosen network output classes—binary detection (e.g., delphinidae vs. noise) or multi-class classification (killer whale vs. white-sided dolphin vs. bottlenose dolphin vs. noise)—which in turn impacts classification complexity. An adequate identification scenario is therefore determined by the biological use-case and taxonomic depth, in combination with the available data material, recorded via active/passive acoustic monitoring. Regarding the animal corpora, two initial data material situations are possible: (1) dataset only contains background noise and target signals, or (2) dataset includes background noise, target signals, and other vocalizations that often resemble the target signal. Consequently, the following classification procedures are conceivable: (1) binary target/noise detection—isolating environmental noise from the taxonomic-dependent animal signals according to the above mentioned data scenarios, or (2) multi-class species/call type recognition—classifying between multiple target species or call types, combined with the illustrated potential data situations. To ensure a robust, animal-independent identification procedure, a binary target/noise detection at the desired taxonomic level (e.g., animal genus) has to be conducted first, to remove noise and other irrelevant animal vocalizations in advance. Taxonomic depth leads to an increasing spectral closeness between signals being represented in the noise class, which results in less distinctive network features separating both classes. Depending on the initial model performance for the chosen taxonomic rank, target/noise data distribution might be restructured with respect to a higher, more generic taxonomic level (e.g. genus to order-level). Based on the respective detection result, subsequent multi-class classification can be conducted with respect to more specific taxonomic ranks, such as animal species (e.g., Blue-winged vs. Golden-winged warbler) or different call types (e.g., monk parakeet alarm, contact, and other calls), ending up in a multi-stage classification procedure. ANIMAL-SPOT is also capable of performing recognition with respect to different species-specific regional differences (dialects) as well as individual identification. Sufficient representative data for dialects of interest or individual-specific vocalizations, in the same way as for the other multi-class classification problems described here, is the only precluding factor. Filtering away noise and other animal vocalizations via the two-step approach enables focus on analysis of regional differences (dialects) and acoustic identification of individuals. Instructions on model configuration and necessary data structure will be further detailed in the user guide^33^ with examples.

### Animal species and recording setup

In order to show and prove animal independence, 10 different species and 1 additional genus within the chordate phylum were chosen. The overall goal was to test model robustness for as many different habitat types (urban parks, marine reserve, arctic landice), frequency ranges (30 Hz for Atlantic cod to 100,000 Hz for Pygmy pipistrelle), vocalization durations (echolocation sweeps in ms to long roars of multiple seconds), signal-to-noise ratios (urban parks versus noise isolated laboratory), noise characteristics (underwater noise, human narrations, other species), as well as recording setups (passive acoustic monitoring—e.g., Harbour seals—versus focal follows—e.g., Blue-/Golden-winged warblers). A detailed summary regarding all animal-specific recording and data collection setups, utilized within this study, is given in Supplementary Table S1.

### Bioacoustic data material

No animals were approached for this study specifically but rather all data used were collected by distinct research teams under their own ethics guidelines. In case of binary detection, each species/genus *target* and *noise* was manually annotated. The *target* class contained only vocalizations produced by the target species/genus (see Table 1). In cases where further sub-classification was envisioned (species level for the two warblers—Table 2, call type level regarding monk parakeets—Table 3), these were labeled as well, but all assigned to the *target* class. The *noise* class included all other sound segments, such as environmental/background noise, human narrations, and other animal sounds. While both the number of annotated segments and the class distribution differed for each species, the ratio between vocalization and noise ranges from $\approx$20% up to $\approx$57% for all listed data archives. To perform embedded noise augmentation, additional noise segments were provided for some of the species (see Table 1). ANIMAL-SPOT was trained and evaluated in three different experiments: (1) detection between *target* and *noise* to separate noise from valuable animal signals, and (2) multi-class species classification, and (3) multi-class call type identification. Besides the annotated detection data corpora, reported in Table 1, three additional unseen recordings were provided for the 10 different species and 1 extra genus, with low, medium, and high appearance of target vocalizations. These were additionally used to validate model performance. In order to prove detection accuracy even further, an additional publicly-available dataset was utilized—the BirdVox-Full-Night data archive—presented by Lostanlen et al.^36^ for the evaluation of approaches designed to detect avian flight calls (see Table 1, last row). The original dataset consists of 9.8 h of audio, recorded by six sensors placed in the area around Ithaca, New York, which were manually annotated resulting in 35,402 500 ms-long flight calls of nocturnally migrating birds of about 25 species of passerines. To balance the dataset an equal number of 500-ms-long noise samples were added to the dataset, resulting in 70,804 files (see Table 1, last row). Regarding the BirdVox-Full-Night archive, there were no additional unseen recordings, compared to the remaining data repositories listed in Table 1. Multi-class species classification was conducted between Blue-winged and Golden-winged warbler (see Table 2). In addition the Computational Paralinguistics Challenge Primate (ComParE-PRS)^37,38^ dataset was used to distinguish between four different primate species (see Table 4). The dataset includes over 10,000 annotated vocalizations from Chimpanzees (*Pan troglodytes*), Mandrills (*Mandrillus sphinx*), Red-capped mangabeys (*Cercocebus torquatus*), and a mixed group of Guenons (*Cercopithecus* spp.). Additionally, exactly the same number of noise samples as vocalizations were extracted to make up the noise class^38^. Multi-class call type classification was computed for three main call type classes of monk parakeet vocalizations including alarm, contact, and other calls, listed in Table 3. Across all multi-class scenarios, the existing target class repertoire was extended by an additional noise category to simulate real-world scenarios, as well as cover and handle potential false alarms caused within the first detection stage (see Supplementary Figs. S5 and S6).

**Table 1:** Animal-specific data corpora and distribution.

Dataset	Label type											
	Target label				Noise label				$\sum$ labels			
	smp[#]$^{1}$	smp[min.]$^{2}$	smp[%]$^{3}$	$\sum$r[min.]$^{4}$	smp[#]$^{1}$	smp[min.]$^{2}$	smp[%]$^{3}$	$\sum$r[min.]$^{4}$	smp[#]$^{1}$	smp[min.]$^{2}$	smp[%]$^{3}$	$\sum$r[min.]$^{4}$
Cockatiel*	1271	12.41	40.9	2.46	1840	41.68	59.1	179.10	**3111**	54.09	100.0	181.56
Sulphur-crested cockatoo	1495	15.26	41.1	3.37	2145	34.99	58.9	46.81	**3640**	50.25	100.0	50.18
Peach-fronted conure	1174	12.35	55.2	0.22	952	8.88	44.8	4.53	**2126**	21.23	100.0	4.75
Monk parakeet*	3133	17.20	46.4	0.63	3612	75.25	53.6	12.63	**6745**	92.45	100.0	13.26
Blue-/golden-winged warbler*	1616	48.99	32.0	5.43	3431	95.10	68.0	14.57	**5047**	144.09	100.0	20.00
Chinstrap penguin	906	4.86	20.8	0.82	3454	15.40	79.2	3.26	**4360**	20.26	100.0	4.08
Atlantic cod	382	3.14	30.6	0.19	867	6.00	69.4	20.82	**1249**	9.14	100.0	21.01
Harbour seal*	2900	55.18	56.4	7.79	2245	58.67	43.6	22.21	**5145**	113.85	100.0	30.00
Killer whale^34^*	17,104	649.45	27.8	20.64	44,323	2076.36	72.2	121.49	**61,427**	2725.81	100.0	142.13
Pygmy pipistrelle*	1570	0.18	31.0	0.10	3490	4.94	69.0	1.11	**5060**	5.12	100.0	1.21
Chimpanzee*	7079	231.17	57.2	2.89	5305	174.44	42.8	87.11	**12,384**	405.61	100.0	90.00
BirdVox-Full-Night^36^	35,402	295.02	50.0	−	35,402	295.02	50.0	−	**70,804**	588	100.0	−

*Additional noise augmentation training samples:

[1.1] cockatiel—180 (2.87 min.), [1.2] monk parakeet—105 (1.08 min.), [1.3] Blue-/Golden-winged warbler—500 (10.03 min.), [1.4] Harbour seal—2,531 (32.74 min.), [1.5] killer whale—6715 (258.27 min.), [1.6] Pygmy pipistrelle—543 (1.80 min.), [1.7] chimpanzee—1446 (40.34 min.).

^1^Samples (smp[#]), ^2^sample duration in minutes (smp[min.]), ^3^sample percentage (smp[%]), ^4^summed target/noise duration of the three unseen test recordings ($\sum$r[min.]).

Significant values are in bold.

**Table 2:** Blue-/golden-winged warbler data distribution.

Label type	Distribution		
	Samples	Min.	%-samples
Blue-winged warbler	707	21.16	22.4
Golden-winged warbler	909	27.83	28.8
Other bird	542	13.39	17.2
Noise	1000	26.10	31.6
$\sum$	**3158**	88.48	100.0

Significant values are in bold.

**Table 3:** Monk parakeet call type data and distribution.

Label type	Distribution		
	Samples	Min.	%-samples
Alarm call	798	5.61	24.5
Contact call	689	3.61	21.2
Other call	764	3.06	23.5
Noise	1000	25.65	30.8
$\sum$	**3251**	37.93	100.0

Significant values are in bold.

**Table 4:** The INTERSPEECH 2021 Computational Paralinguistics Challenge Primate (ComParE-PRS)^37,38^ data archive and distribution.

Label type	Distribution		
	Samples	Min.	%-samples
Chimpanzee	6652	59.76	32.0
Mandrills	2623	13.14	12.7
Red-capped mangabeys	627	5.31	3.0
Guenons	476	1.69	2.3
Noise	10,378	172.97	50.0
$\sum$	**20,756**	252.87	100.0

Significant values are in bold.

### Deep learning concepts and network architectures

Convolutional Neural Networks (CNNs) were utilized in order to identify animal vocalizations of interest. A CNN is an end-to-end deep learning architecture based on the principles of pattern recognition including a feature learning and classification component being able to efficiently process the complexity of 2-dimensional input data (e.g., images, spectrograms)^34,40,41^. Convolutional layers are responsible for feature learning, while the classification part is done by the fully connected layers^40^. Convolutional layers embed and represent the following important concepts^34,40^: (1) local receptive fields, (2) shared weights, and (3) subsampling (pooling). Due to the fact that convolutional and pooling layers only compute linear operations, CNNs integrate activation layers (e.g., Rectified Linear Unit^34,42^) as well as normalization layers (e.g., batch normalization^34,43^) to handle the non-linearity within the data and to ensure a more stabilized and regularized training procedure^34^. Several repetitive sequentially ordered sequences of convolutional, pooling, normalization, and activation layers lead to extracted and learned features which are used as input for the fully-connected layer projecting the features on the respective output classes^34^. The core concept of the presented deep learning framework is based on a so-called Residual Network (ResNet)^35^. A ResNet is a network architecture, which is built up from different concatenated residual layers^35^. A residual layer is constructed from an arrangement of building blocks which in turn consist of weight (e.g., convolutional, fully-connected), normalization (e.g. batch-norm^43^), and activation layers (e.g., ReLU^42^), as well as residual-/skip-connections. Due to this residual-/skip-connection technique it is possible to learn a residual mapping $F(x)=H(x)-x$ instead of a direct underlying mapping *H*(*x*) for a given input *x*^35^, enabling to counteract the accuracy degradation problem (accuracy decrease after saturation region, by further increasing network depth, compared to shallower versions of the network^35^) and training deeper nets. Different numbers and structures of building blocks result in various ResNet architectures. Well known and established ResNet models are ResNet18, ResNet34, ResNet50, ResNet101, and ResNet152^35^. For more detailed insights about residual learning/networks see He et al.^35^.

### ANIMAL–SPOT

**Figure 2:**
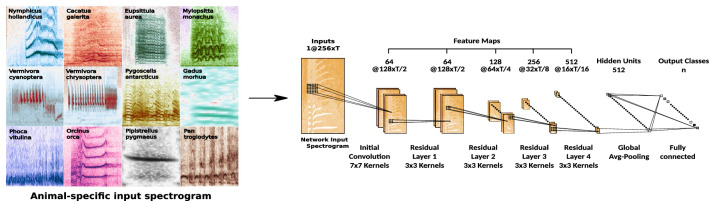
ANIMAL-SPOT Network Architecture (created via Inkscape^39^, Version 0.92.3).

The deep learning framework consists of a ResNet18-based CNN, derived from ORCA-SPOT^34^, our previous killer whale deep detection model, which has been adapted and extended to handle all kinds of vocalizing animals. The initial max-pooling layer within the traditional ResNet18 architecture has been removed to avoid losing too much resolution at the early stage of the training process^34^. Depending on the size of the temporal domain *T* of the input spectrogram, defined by the chosen training sequence length and corresponding FFT-settings, a 512-large global-averaged pooled feature vector, derived from the 512 × F × T feature maps of the last residual layer (see Fig. 2), is generated and mapped to a subsequent fully-connected layer^34^. In order to solve the final n-class classification problem the 512 hidden units of the fully-connected layer are processed onto an output layer consisting of *n* output nodes depending on the classification task (e.g., two classes for target/noise detection, or multiple classes for species/call type classification). ANIMAL-SPOT is capable of handling any number of output classes, and consequently dealing with multi-class classification scenarios as well. Moreover, ANIMAL-SPOT integrates a refactored version of the entire data parsing and pre-processing pipeline of ORCA-SPOT^34^, next to additional normalization techniques, in order to handle and fulfill all needed prerequisites required for dealing with various animal data sources (see Fig. 1 and Tables 1, 2, 3, 4), in combination with varying classification scenarios. Although advances in neural network structures have been made in recent years, the focus of ANIMAL-SPOT is not a specific type of architecture (e.g., ResNet18, etc.). Instead, the aim of ANIMAL-SPOT is to provide an open-source, animal-independent, and expandable machine learning framework, together with a robust and efficient data preprocessing pipeline. We add support by profound user guidelines to address the broadest possible audience. In addition, the capacity of the model used here is comparatively small, which facilitate researchers who may not have the opportunity to access powerful hardware, to train and evaluate their own animal- and task-specific models. Within the ANIMAL-SPOT framework it is possible to integrate any kind of architectural model designs, allowing the deployment of other novel and user-preferred deep neural network concepts.

### Data preprocessing

**Figure 3:**
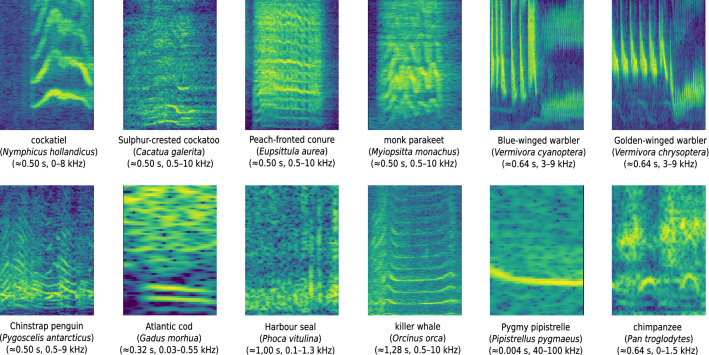
ANIMAL-SPOT preprocessed 256 × 128-large network input spectrograms (256 frequency bins, 128 time frames) utilizing animal-specific network-hyperparameters listed in Supplementary Table S2 (created via Inkscape^39^, Version 0.92.3).

Independent of the classification scenario all species and their corresponding data repositories (see Tables 1, 2, and 3), followed the same generic data preprocessing pipeline. The core functions are applicable for all animals, however each species requires an animal-specific parameter set (see Supplementary Table S2) in order to guarantee valid data preparation and representation of the corresponding signal characteristics (e.g., typical vocalization duration, frequency range, sampling rate, Fast-Fourier-Transform (FFT) parameters, etc.). The entire preprocessing pipeline of ANIMAL-SPOT consists of the following steps: (1) conversion to mono and re-sampling, (2) Short-Time-Fourier-Transform (STFT) to convert the time signal into a F × T-large power-spectrogram using an animal-characteristic FFT window-length and step-size, where *F* characterizes the frequency domain and *T* describes the time domain, (3) integrated and concurrent signal augmentation with respect to the previous derived F × T-large power-spectrogram applying uniformly distributed random scalings including intensity, pitch, and time augmentation within given intervals (see Supplementary Table S2), where the default interval might slightly vary from species to species, (4) linear frequency compression (nearest neighbor, 256 frequency bins) representing a frequency range between $f_{min}$ and $f_{max}$, while ignoring other frequency regions, chosen according to the typical spectral vocalization areas of the corresponding animals, resulting in a 256 × T compressed power spectrogram, (5) noise augmentation by adding a pitch-/time-augmented and frequency-compressed noise spectrogram utilizing a uniformly distributed randomly chosen signal-to-noise ratio (SNR), (6) power-spectrogram conversion to decibel (dB) scale, (7) 0/1-min/max- or 0/1-dB-normalization, either using the spectral minimum and maximum, or applying a minimum and reference decibel level, dependent on the respective target species, to normalize the spectral envelope, and (8) random sub-sampling or zero-padding of the spectrogram according to the chosen sequence length, leading to a final 256 × T augmented and normalized spectral clip being used as network input. Figure 3 visualizes example network input spectrograms for each species, preprocessed according to the illustrated pipeline.

### Network training and evaluation

Due to ANIMAL-SPOT’s ResNet18-based feature extraction and compression path (see Fig. 2), each input spectrogram is compressed by a factor of 16 during encoding, both in time *T* and in frequency *F* domain. The remaining F × T features for each of the 512 channels are mapped to the corresponding fully connected layer, conducting global average pooling, followed by a projection to the number of parametrizable output nodes/classes. During training, random data augmentation and sub-sampling/padding, can be enabled. However, to compare validation and test set results across various models, random data augmentation and sub-sampling/padding was disabled. Validation and test samples were centered and either zero-padded or sub-sampled, in case the original length did not match the chosen sequence length^34^. ANIMAL-SPOT was implemented in PyTorch^17^ using a cross entropy loss in combination with a batch-size of 8 for all animals, together with an Adam optimizer applying an initial learning of $10^{-5}$, $\beta _{1}$ = 0.5, and $\beta _{2}$ = 0.999. Additionally, ANIMAL-SPOT integrates a learning rate decay of 1/2 after 4 epochs without any improvement on the validation set. The training was stopped after an animal-specific number of epochs (see Supplementary Table S2) if no improvement was achieved on the validation set (early stopping). The accuracy was chosen as an appropriate network validation criterion. ANIMAL-SPOT integrates an intelligent data split mechanism, capable of automatically identifying all class labels, assuming that data preparation was performed in the prescribed format^33^, and ensures that samples of a particular recording are only present in one of the splits. By default, the data split is 70% for training, 15% validation, and 15% test. However, it may differ depending on the original data distribution in combination with the above mentioned recording restriction (see Supplementary Tables S4–S6). Regarding the two challenge datasets—BirdVox-Full-Night^36^ and ComParE-PRS^37,38^—the original predefined data splits have been applied for reasons of comparison. Network training and evaluation was computed utilizing mid-range graphics processing units (GPUs) (e.g., Nvidia GTX 1080), as well as standard central processing units (CPUs), showing the broad applicability of the training setup. Supplementary Table S2 reports all animal-specific network-hyperparameters.

ANIMAL-SPOT’s network performance was evaluated via the following experimental constellations: (1) animal- and scenario-specific model evaluation for target/noise detection (see Supplementary Table S3) and multi-class classification (see Supplementary Tables S4, S5, S6), reporting various performance metrics regarding training, validation, and unseen test set, (2) evaluation of animal-specific target/noise detection networks on three fully-annotated unseen test recordings, performing a sliding window approach in combination with a given window-length $\epsilon$ and step-size $\kappa$ to frame-wise segment between target and noise, and (3) inspection and verification of the multi-class classification models for warblers and monk parakeets based on the machine-segmented and extracted signal parts of step 2, generated by the corresponding segmentation models.

The first evaluation scenario visualizes the following training, validation, and test metrics: accuracy (ACC), true-positive-rate (TPR), false-positive-rate (FPR), precision (PREC), F1-score (F1), and area under the ROC curve (AUC). In case of the ComParE-PRS^37,38^ primate species recognition challenge, only the unweighted average recall (UAR) was reported due to comparability reasons.

The second evaluation procedure classifies audio sections for each of the three unseen, animal-specific test recordings, depending on the defined window-length $\epsilon$ and step-size $\kappa$, affecting the signal overlap, as a whole. Machine-predicted audio chunks are compared frame-by-frame with the ground truth^34^. Each predicted frame/segment of the unseen recordings, together with the respective frame-wise network confidence (probability), allow to present the ROC-curve^44^ and its respective AUC^34^. In addition, frames showing a larger value than a model confidence $\delta$, are transformed into an annotation with its corresponding start and end time. Therefore, successive frames of the same label (noise = 0 or target = 1) are concatenated and extracted as one annotation excerpt^34^. Frame-wise smoothing was used to mark classified noise segments as target frames if the neighboring signal chunks are exclusively labeled as target signals^34^. Neighboring frames are frames which include preceding or subsequent signal content of the current sound segment because of the respective overlap^34^. ANIMAL-SPOT-S refers to the smoothed version, whereas ANIMAL-SPOT corresponds to the non-smoothed variant (see Supplementary Figs. S2–S12). Additionally, the predicted, smoothed, and extracted network detections with an exemplary model confidence of $\delta \ge \,50\%$ and $\delta \ge \,90\%$, were used to calculate and report time-wise precision (PREC) versus corresponding recall (TPR), in order to show intersection accuracy between machine- and human-annotated labels. To calculate time-based precision and recall all ground truth annotations, which are not further apart than a merging factor $\xi$ = $\frac{\epsilon }{2}$ (half of the prediction window in seconds), were combined to one annotation, since such cases lead to sliding windows $\epsilon$, while at least half of the window contains animal vocalizations. In case of time-wise precision calculation an additional overlapping factor $\lambda$ = $\frac{\epsilon }{2}$ was introduced, extending the ground truth annotation start and end accordingly (start − $\lambda$, end $+$ $\lambda$), covering overlapping predictions at the annotation borders.

**Figure 4:**
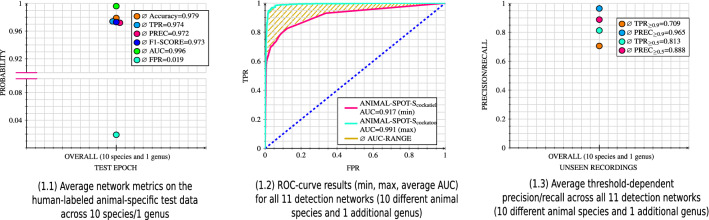
Overall summary across all 11 animal-specific segmentation models (10 different species, 1 additional genus), visualizing performance metrics with respect to the animal-specific, unseen, human-labeled test data (**1.1**), ROC-curves and AUC-Range (**1.2**), as well as threshold-dependent precision/recall values (**1.3**), both based on the animal-related unseen recording tapes (see also species-specific results in Supplementary Figs. S2–S12 and Supplementary Table S7) (created via Inkscape^39^, Version 0.92.3).

**Figure 5:**
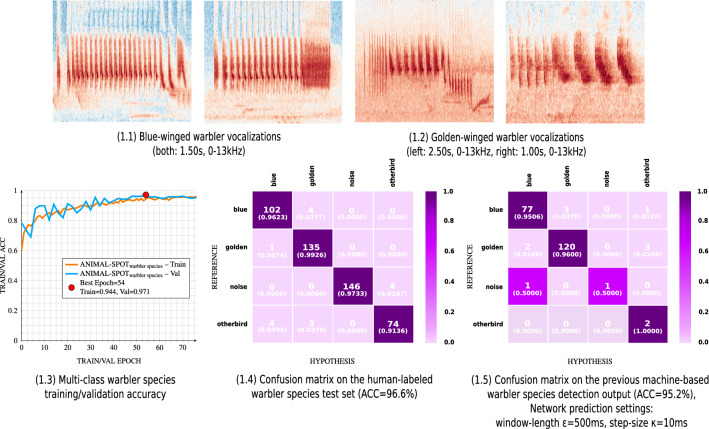
Multi-class warbler species identification results, visualizing spectrogram examples of Blue-/Golden-winged warblers (**1.1**,**1.2**), multi-class training/validation accuracy (**1.3**), confusion matrix regarding the unseen human-labeled test data (**1.4**), as well as confusion matrix concerning previous machine-based warbler detection (**1.5**) (created via Inkscape^39^, Version 0.92.3).

**Figure 6:**
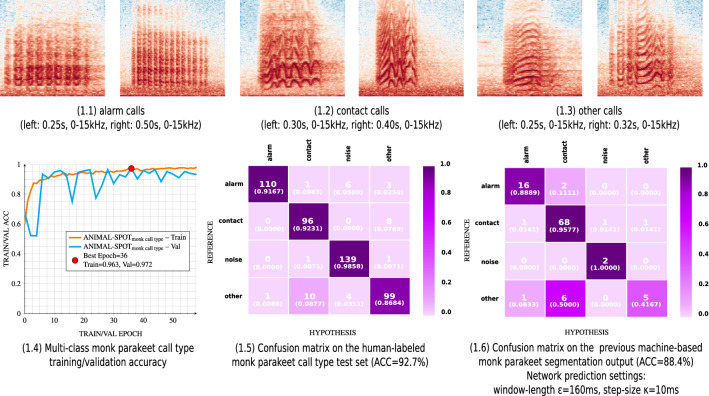
Multi-class call type classification results, visualizing spectrogram examples of alarm, contact, and other call types (**1.1**–**1.3**), multi-class training/validation accuracy (**1.4**), confusion matrix regarding the unseen human-labeled test data (**1.5**), as well as confusion matrix concerning previous machine-based monk parakeet detection (**1.6**) (created via Inkscape^39^, Version 0.92.3).

The third and last evaluation scenario reports results on multi-class classification by presenting the following evaluation criteria on training, validation, and test data: (1) accuracy, (2) confusion matrix, and (3) UAR (only for the ComParE-PRS^37,38^ dataset). Additionally, the model was evaluated on the corresponding machine-annotated warbler and monk parakeet results, utilizing the same sliding window approach. However, during prediction of the multiple classes, a noise identification was only considered as correct if the network confidence was higher than > 85%, due to the assumption that the previous detection process has a low false positive rate. If the network’s confidence regarding noise was lower than this boundary, but still the highest probability, it was ignored and the second largest confidence value was chosen as correct prediction. The chosen window length, in combination with the input file duration, ends up in two potential cases: (1) window length is larger than the input file duration, leading to an updated window equal to the pre-segmented audio clip, and (2) window size is shorter than the input file size, leading to a sliding, frame-wise classification approach determined by window- and step-size, while only considering full windows in order to avoid potential misclassification. If multiple sequentially-ordered frame-wise classifications per pre-segmented file exist, probabilities of each frame and predicted class are summed up cumulatively. Finally, the class providing the largest probability mass was selected. All detection and multi-class species/call type classification metrics are visualized and illustrated in Figs. 4, 5, 6, as well as Supplementary Table S7 and Supplementary Figs. S2–S12.

### ANIMAL-SPOT guide

The ANIMAL-SPOT Guide^33^ is a step-by-step and detailed user guide, publicly available together with the source code^33^, which enables researchers to train and evaluate animal-specific deep neural networks on their own bioacoustic data corpora (see Supplementary Fig. S1). The guidelines involve: (1) operating-system independent installation, data preparation, and detailed documentation of the ANIMAL-SPOT source code^33^, (2) instructions and guidance in order to set up, train, and evaluate animal- and scenario-specific architectures, as well as (3) use-case dependent prediction of unseen data material utilizing stand-alone noise/target detection models, species/call type classification networks, or a combined version of detection and subsequent classification (see Supplementary Fig. S1). The ANIMAL-SPOT guide provides a detailed description with respect to the following three scenarios: (1) single-stage detection between animal vocalizations of interest and noise, based on unseen data, (2) single-stage classification of animal species and/or specific call types directly on unseen raw audio material, and (3) a combined version of step 1 and 2 by firstly pre-segmenting unseen audio recordings, followed by subsequent classification (animal species, call types, etc.), while taking only the respective pre-segmented target vocalizations as input.

## Experiments

### Animal-species target/noise segmentation

In a first experiment animal-species segmentation was performed for all animal-specific (see Fig. 1) data volumes listed in Table 1. Data partitioning was conducted for each animal-specific data archive (see Supplementary Table S3), whereas the training set comprises $\approx$70%, validation and test set each $\approx$15% of the total labeled data corpora listed in Table 1. Using the respective data distributions in combination with the animal-specific network-hyper-parameters presented in Supplementary Table S2, different ANIMAL-SPOT architectures were trained and evaluated according to the previously described network training and evaluation procedure. An exception is the BirdVox-Full-Night^36^ challenge dataset (see Table 1, last row), which used the same data distribution, training, and evaluation procedure as described in Lostanlen et al.^36^, in order to allow a meaningful comparison with the original results. Consequently, a leave-one-out testing procedure whereby one unit of the given six was utilized for testing and the other five were used for training and validation. This results in exactly the same data split as reported, but also means that there exist no additional and unseen data available for further evaluation, as it was the case for all other data corpora listed Table 1. For reasons of comparison and the sake of completeness, parts of the already published results on killer whales (see ORCA-SPOT^34^) are reported and visualized as well.

### Multi-class species and call type classification

As baseline for the second experiment, results of the first target/noise (binary) animal-species segmentation were utilized, next to a pure stand-alone multi-species classification scenario without pre-segmentation. To demonstrate, prove and verify performance of the proposed multi-step classification procedure, three of the 12 animal species (see Fig. 1) were utilized—Golden-/Blue-winged warblers (genus) and monk parakeets.

In case of the warblers a subsequent multi-class classification model, trained on the data and distribution listed in Supplementary Table S4, was used to further separate the genus-specific and previously machine-segmented data pool into Golden-winged, Blue-winged warblers, other birds, and noise. To counteract possible false alarms from the segmentation phase, two classes—other birds and noise—were introduced besides the corresponding signals of interest.

The proposed multi-stage approach was further evaluated training a multi-class classification network to differentiate between various monk parakeet call types, using the data and distribution in Supplementary Table S5, including contact, alarm, and other calls, as well as noise, in order to handle previous segmentation errors.

In case of the golden- and blue-winged warblers, a total of 210 machine-annotated audio segments were extracted utilizing a network confidence of $\ge$ 90%. Under identical conditions 103 monk parakeet machine segmentations were predicted and extracted. Example spectrograms for golden-/blue-winged warbler vocalizations, as well as for the various monk parakeet call types, are visualized in Figs. 5 and 6.

In order to assess the efficacy of the multi-stage approach, instead of a single-stage multi-class approach, a multi-class model was exemplarily trained to perform detection and classification of Golden-winged warbler, Blue-winged warbler, and noise (pure background noise, other birds) in one step, using exactly the same three unseen, manually labeled recordings for evaluation as during the detection phase within the multi-stage procedure (see Table 1). The three audio files contain either: (1) only Golden-winged warblers, (2) only Blue-winged warblers, (3) a combination of both warbler types. The model used the same data distribution for Golden-/Blue-winged warbler as stated in Supplementary Table S4, together with the warbler noise distribution listed in Supplementary Table S3.

Nevertheless, in order to also show and demonstrate the possibility of directly training a multi-species classification network without previous segmentation, the ComParE-PRS dataset (see Table 4) was used to distinguish between 4 different primate species as well as background noise, trained on the given data distribution listed in Supplementary Table S6.

## Results

### Animal-species target/noise segmentation

ANIMAL-SPOT successfully segmented all 10 target species, as well as the additional genus, leading to an overall mean test set accuracy of 97.9% (range: 94.5–99.8%). Additionally, an average area under the ROC curve (AUC) of 95.9% (range: 91.7–99.1%) across all 33 unseen animal-specific recordings (3 tapes per detection scenario) was achieved (see Supplementary Table S7. Besides network generalization on the unseen tapes, a detailed performance overview with respect to model training, validation, and testing is reported and visualized in Supplementary Figs. S2–S12. In addition, all detection results are summarized and available in Supplementary Table S7. Moreover, Fig. 4 summarizes detection results in a compressed version, averaged across all 11 animal-specific segmentation models (10 different species, 1 additional genus), visualizing: (1) network performance metrics based on the animal-specific human-annotated training, validation, and testing repositories (see Supplementary Table S3, Fig. 4—1.1), (2) model results across all 11 averaged Receiver-Operating-Characteristics^44^ (ROC) curves by visualizing 2 out of 11 curves, indicating the minimum and maximum AUC, spanning the average AUC-range where all other remaining ROC-curves are located (Fig. 4—1.2), and (3) network output across all 11 averaged and threshold-dependent precision/recall scores (Fig. 4—1.3). Apart from the segmentation results of all 11 animal-specific segmentation models (10 different species, 1 additional genus), ANIMAL-SPOT also successfully processed the BirdVox-Full-Night^36^ dataset. In comparison to the results given by Lostanlen et al.^36^, with the best performance coming from a CNN with noise augmentation, which resulted in an average accuracy of 94.9% and an average F1-Score of 62.7%, ANIMAL-SPOT achieved a slightly better average accuracy of 95.4% and a significantly better F1-Score of 95.4%. These results were achieved by training 10 models for each unit and taking the average of the results when removing the best and worst two performing models, resulting in an average over six models for each unit.

### Multi-class species and call type classification

Multi-class species classification was applied to the previous warbler target/noise detection results (see Supplementary Fig. S6), in order to further separate the machine-segmented warbler species vocalizations, exemplarily visualized in Fig. 5—1.1,1.2, into Blue-winged and Golden-winged warbler, resulting in a multi-class (4-classes) species identification scenario. Therefore, ANIMAL-SPOT, trained in a multi-class species classification scenario using the data listed in Supplementary Table S4, achieved an overall accuracy of 96.6% for the human-labeled unseen test set, as well as 95.2% with respect to the total number of 210 previously machine-detected audio segments (see Supplementary Fig. S6). Moreover, training and validation accuracy is shown besides two confusion matrices (4 classes), visualizing the aforementioned results achieved on the respective unseen human-labeled and machine-segmented test corpora (see Fig. 5—1.3–1.5, Supplementary Fig. S6).

Compared to the results of the proposed two-stage approach, which includes target/noise detection and downstream multi-class identification, the single-stage method, which performs both, detection and classification, in a multi-class model at once, performed significantly worse. Across all three unseen warbler recordings the Golden-/Blue-winged warbler detection model (threshold $\delta$ $\ge$ 0.9) identified a total of 210 potential vocalizations of interest, resulting in a time-based precision of 95.3% (see Supplementary Fig. S6). All 210 of the segmented samples were then used for downstream multi-class classification. In comparison, the 3-class single-stage approach detected 233 warbler events, with 200 true predictions resulting in a sample-based precision of 85.8%, whereas just 77.3% (180 out of 233 vocal events) were detected and classified as the correct warbler species. In the case of the two-stage approach multi-species classification achieved an accuracy of 95.2% (see Fig. 5). This indicates that a two-step approach, where the network can focus more on the distinguishing features of the individual bird species without also having to filter out as much noise, is preferable to a single-step approach.

Besides all the results regarding warbler-species classification, ANIMAL-SPOT was also successfully deployed to identify various primate species using the Computational Paralinguistics Challenge Primate (ComParE-PRS)^37,38^ data archive. The initial challenge investigation utilizes five different approaches for feature extraction and classification of primate vocalizations, namely openSMILE^45^, openXBOW^46^, DeepSpectrum^47^, AuDeep^48^, and End2You^49^ in conjunction with either an SVM (openSMILE, openXBOW, DeepSpectrum, AuDeep) or a recurrent neural network (RNN) (End2You) for the final classification. The initial baseline for the challenge, calculated by majority voting using the best configuration for each approach, reported the best unweighted average recall (UAR) of 87.5%^37,38^. In comparison, ANIMAL-SPOT outperformed the baseline achieving a UAR of 89.3%. Multi-class call type classification was applied to the previous monk parakeet target/noise detection results (see Supplementary Fig. S5), similarly to the warblers, however, with the aim to classify between different call types visualized in Fig. 6—1.1–1.3, leading to a multi-class (4 classes) monk parakeet call type classification scenario. For this purpose, ANIMAL-SPOT was trained on the data listed in Supplementary Table S5. The final model achieved an overall test set accuracy of 92.7%, compared to 88.4% on the previous machine-based detection results (see Fig. 6—1.4–1.6, Supplementary Fig. S5).

## Discussion and future outlook

In total, 10 different species and 1 extra genus (see Figs. 1, 4, Supplementary Figs. S2–S12), as well as the publicly-available BirdVox-Full-Night^36^ dataset, were analyzed in a binary detection scenario in order to prove ANIMAL-SPOT’s ability to generalize across a wide variety of sound-types and to assess the feasibility of the proposed multi-stage detection/classification pipeline (see Figs. 5 and 6). As the results on the unseen recordings prove, promising time-wise and threshold-dependent recall/precision values were achieved, indicating an accurate intersection between ANIMAL-SPOT’s predictions and the actual ground truth (see Fig. 4—1.3, Supplementary Table S7 and Supplementary Figs. S2–S12). In addition, ROC-curves and corresponding AUC values show a significant reduction of the species-dependent and original noise-heavy data material (see Fig. 4—1.2). Thus, threshold-dependent recall and false-positive-rate combinations can be derived according to the respective use-case, which in turn considerably speeds up and improves downstream data analysis. Furthermore, the combined strong results seen in both unseen test set as well as unseen real-world recordings, suggest no indication of model overfitting and prove network generalization across all different animal species.

The improvements with respect to the publicly available BirdVox-Full-Night^36^ dataset are also very promising, as the detection accuracy was improved by 0.6%, which indicates an error reduction of about 12%, besides a significant improvement of 32.7% regarding the F1-Score.

ANIMAL-SPOT integrates a large repertoire of distinct parameterization options for setting up data preprocessing and network training (see Supplementary Table S2). Thus, ANIMAL-SPOT performs equally across wide ranges of temporal contexts (e.g average vocalization duration of Pygmy pipistrelles compared to killer whales), frequency ranges (e.g low-range Atlantic cod and Harbour seal vocalizations, mid-range bird sounds, and ultrasound bat signals), as well as spectral patterns (e.g., pulse-like structure of the Harbour seal or warbler signals and harmonic properties of the killer whale, Atlantic cod, and chimpanzee vocalizations). It is even possible to learn and distinguish between spectral call structures which are very similar to noise, seen in ANIMAL-SPOT’s exemplary ability to distinguish Sulphur-crested cockatoo, Harbour seal, monk parakeet, and chimpanzee vocalizations from very similar background noise. In case of binary target/noise detection, ANIMAL-SPOT is especially useful in recording situations where the noise characteristics are an order of magnitude larger than the amount of valuable animal vocalizations.

ANIMAL-SPOT’s parameterization capacity also enables flexible adaptations regarding model architecture, data preprocessing, and network training/evaluation, allowing researchers to address and answer various specific bioacoustic research questions. Furthermore, the binary-class target/noise detection process enables researchers to separate target species that show poor results in the single-stage binary target/noise detection scenario. This can occur especially when the target species spectrally resemble other vocalizing species that are also found in the unseen recordings. In such situations the primary focus is on a generic distinction between target vocalizations and superfluous noise, making subtle spectral differences of other species difficult to model, because of generalization properties across both classes leading to increasing mis-classifications. This phenomenon was observed in case of Blue-winged and Golden-winged warblers, after both were individually trained and analyzed on species level, which demonstrated significant performance variations. However, using a two-step identification scenario consisting of target/noise detection at genus level (see Supplementary Fig. S6), and subsequent multi-class species classification, ANIMAL-SPOT achieved an overall test set accuracy of 96.6% on unseen test data, which had been labeled by a human expert, and an accuracy of 95.2% on the target detections identified by ANIMAL-SPOT in the target/noise detection scenario (see Fig. 5).

In addition, the same two-step approach was successfully applied to distinguish and classify between different monk parakeet call types, resulting in 92.7% test set accuracy for human-annotated samples, and 88.4% with respect to the machine-performed detection results (see Supplementary Fig. S5, see Fig. 6). These combined results demonstrate the wide range of biological scenarios which can be covered by ANIMAL-SPOT in combination with user- and animal-specific data material. In both multi-class classification scenarios—warbler species and monk parakeet call types—ANIMAL-SPOT extracts and classifies centered signal sections of the unseen network test set samples according to the training sequence length (see Supplementary Table S2, see Figs. 5—1.4 and 6—1.5). However, the pre-segmented audio chunks, different in length, were classified by utilizing a sliding window approach, together with the corresponding settings (see Figs. 5—1.5 and 6—1.6). At each frame the maximum probability of all classes was chosen. The class showing the highest probability mass across the entire signal was selected as the final network hypothesis. In both experiments, each of the confusion matrices show comprehensible and similar results, being an auspicious indicator for model generalization across various data (see Figs. 5—1.4,1.5 and 6—1.5,1.6). Furthermore, sequence length and step-size are very important parameters to guarantee robust predictions. In terms of species and/or call type classification the step-size should be an order of magnitude smaller than the sequence length, in order to guarantee sufficient overlap and not to miss important spectral features during the prediction phase.

ANIMAL-SPOT demonstrated also great results in terms of single-stage multi-species primate classification, by outperforming the ComParE baseline system. The final result of 89.3% also exceeds the UAR of 88.3% presented by challenge competitor Illium et al.^50^, who applied a vision transformer to the classification problem. Müller et al.^51^ report the same UAR of 89.3% while applying a Deep Recurrent Neural Network. The remaining competitors who performed better than ANIMAL-SPOT utilized either ensembling of multiple classifiers, as in the case of Egas-López et al.^52^, who achieved a UAR of 89.8%, or data augmentation techniques such as SpecAugment or MixUp and training tricks such as exponential moving average of the model weights, as presented by Thomas Pellegrini^53^, who achieved a UAR of 92.5% on the test set. ANIMAL-SPOT is therefore placed squarely in the middle of the top challenge performers despite using only a single, relatively simple classifier and basic augmentation techniques.

**Figure 7:**
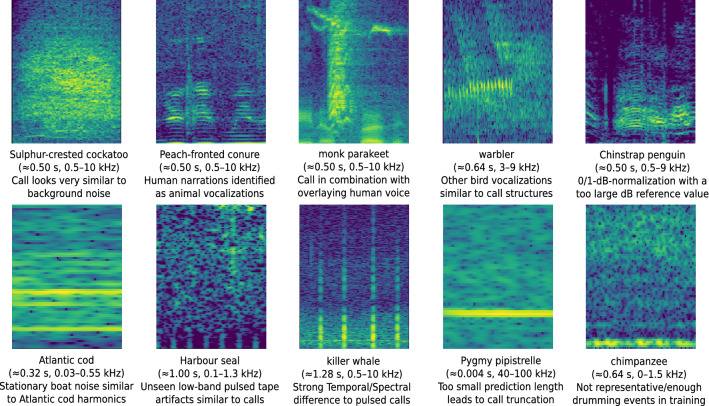
Spectrogram examples visualizing potential error sources leading to performance drops of ANIMAL-SPOT (created via Inkscape^39^, Version 0.92.3).

In order to robustly train and report promising results, data volume, distribution, and variation is crucial. Moreover, the data corpus must be representative with respect to unseen real-world data. If these criteria are not fulfilled, models often lead to significantly worse results, despite promising training, validation, and test metrics. In order to enlarge data variation, especially for small animal corpora, various embedded spectral augmentations were computed (see Supplementary Table S2). However, such augmentation variants and corresponding values must be determined independently for each animal species and can therefore not be generalized. In particular, noise augmentation must be applied carefully, because of differing Signal-to-Noise-Ratio (SNR) between the original sounds and utilized noise data, particularly in case of animal vocalizations being very similar to noise data (e.g., Sulphur-crested cockatoo, distant chimpanzee pant-hoot versus bird vocalizations in the same frequency range). Therefore, it is essential to ensure that noise samples, chosen for augmentation, are representative and independent from training, validation, and test noise excerpts. Despite promising scenario- and animal-specific results on the unseen test data, audio recordings, machine-driven pre-detections, and challenge datasets (see Figs. 4, 5, 6, Supplementary Figs. S2–S12, and Supplementary Table S7), the performance may still vary to a certain extent, due to the following reasons: (1) non-representative data and/or insufficient training data, (2) recording artifacts introducing spectral outliers which are difficult to interpret by the network, (3) other animal vocalizations or noise characteristics showing a similar spectral envelope as the target sounds, (4) strong deviation of the signal intensities compared to the chosen reference and minimum dB-values of the 0/1-dB-normalization during training (see Supplementary Table S2), (5) overlapping animal signals and human narrations, (6) vocalization types of a given species which have significant spectral and temporal differences between each other, and (7) window-length $\epsilon$ and step-size $\kappa$ used during prediction phase. Figure 7 visualizes different examples of such animal- and task-specific misclassifications, caused by the previously illustrated error sources, which significantly influence network prediction results.

**Figure 8:**
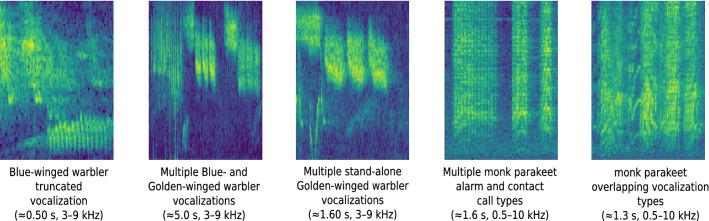
Machine-segmented spectrograms for Blue-/Golden-winged warblers and monk parakeets, visualizing various challenging scenarios for a potential subsequent multi-class classification (created via Inkscape^39^, Version 0.92.3).

Furthermore, during multi-class classification, special attention needs to be paid towards correct machine-based detection outputs indicating one of the following scenarios: (1) multiple vocalizations of the same and/or different species/call types within a single segment, (2) truncated signals, either at the beginning or end of a segment, and (3) overlapping vocalizations. Examples of the above mentioned and remaining challenges are visualized in Fig. 8.

In addition, data collection should be conducted via a consistent recording setup. Including data material originating from varying recording environments and/or setups will result in spurious outputs unless sufficient examples of this variation is represented in the training and validation datasets.

ANIMAL-SPOT’s performance and network training stabilization strongly correlates with the chosen hyper-parameter setup, respective data structure and distribution, as well as model initialization. In order to identify the best fitting training setup for a certain species and classification procedure, a parametric search within the target-specific value range (with regards to e.g., signal frequency range, average sound duration, type of vocalization) should be performed. Additionally suitable prediction settings—window length $\epsilon$ and step-size $\kappa$—as well as network parameters (see Supplementary Table S2) are very important. Window length $\epsilon$ has to be approximately in the same dimension as the network training length. To ensure adequate prediction settings, ANIMAL-SPOT should be evaluated on a small portion of unseen manually labeled recordings, before processing large unseen data archives. Moreover, network initialization, as well as random augmentations during training, may impact network performance, especially in case of small training corpora, affecting final model performance despite similar training, validation, and test set metrics.

Researchers face various acoustic detection scenarios, namely simple target/noise segmentation, identification of target signals among other distinct animal-specific vocalizations, and the recognition of target vocalizations among other similar animal-related vocalizations. All these scenarios can be addressed by the ANIMAL-SPOT framework and its underlying methods. For the simple case of identifying a target signal among nondescript background noise a simple one-step procedure can be applied as well as utilization of the framework-supplied noise augmentation to account for differences in signal-to-noise ratios in varying real-world conditions. Similarly, in the case where the target signal is dissimilar to the to other known vocalizations, a one-step model application procedure can be applied, and the classification is altered from a binary target-noise scenario to a multi-class problem which includes vocalizations from other known species present in the recordings. Finally, when dealing with the scenario in which the target vocalization exhibits similar characteristics such as to make them difficult to discern from each other, a multi-step approach can be taken, as was shown when attempting to accurately distinguish between Blue and Golden-winged warblers (see Fig. 5) or different monk parakeet call types (see Fig. 6). The same applies to the recognition of species-specific dialects and single individuals. The first task is to eliminate to the fullest extent the background noise (pure noise, other dissimilar animal vocalizations) from the classification problem. After background noise is removed from the data, it appears that the model is more capable of distinguishing between similar acoustic features through the focus on other spectral characteristics and features. Note that, due to the relatively small model sizes used here, a two-step approach could also be applied to the case where vocalizations are dissimilar without incurring a significant penalty with respect to computation time.

Besides the animal-independent target/noise and multi-step/class identification results (see Figs. 4, 5, and 6), this study also puts special emphasis on the proposed ANIMAL-SPOT guide^33^ (see Supplementary Fig. S1), which enables researchers to setup their own user-specific deep learning framework, without the need of prior machine-learning knowledge. The ANIMAL-SPOT guide^33^ describes the entire software framework from beginning to end, including OS-specific installation manuals regarding all necessary software components, data preparation and processing guidelines, as well as detailed descriptions on how to setup, train, and run the final network prediction/evaluation on unseen data (see Supplementary Fig. S1).

The entire deep learning framework, as well as user- and animal-specific setup, can be verified and evaluated through the additionally provided example data archive on monk parakeets^54^ , which is publicly available^33^, next to all the source code and user-friendly instruction manual. This guide enables the bioacoustic community to independently train/evaluate task- and animal-specific deep models in order to gain deeper insights into animal communication and understanding.

Many different fields of potential future follow-up work arise, such as (1) animal-specific representation^55^ and/or transfer learning, utilizing larger labeled/unlabeled bioacoustic data corpora and/or other data archives (e.g., ImageNet^56^), (2) investigation regarding various deep network architectures (e.g., CNN-LSTM^25^, ResNeXt^57^, Inception/Inception-ResNet^58^, or Transformer-based approaches^59^), and (3) animal-independent signal enhancement/denoising^60^, acting as additional data preprocessing option. To the best of the authors knowledge, ANIMAL-SPOT is the first open-source^33^ machine learning approach, capable of handling various bioacoustic signal identification scenarios (binary target/noise detection, multi-class species/call type classification), verified on a wide portfolio of animal vocalizations from different animal taxa and challenge datasets. In combination with a detailed user guide, ANIMAL-SPOT allows the broader bioacoustic research community to develop their own task-specific deep neural networks, on virtually any animal species.

## Supplementary Information

Supplementary Information.

## Data Availability

The acoustic data archives supporting the findings of this study are available from the respective data owners upon reasonable request. Contact details can be obtained from the corresponding author. Upon acceptance, the code for ANIMAL-SPOT, besides the entire ANIMAL-SPOT guidelines, all together with an example data corpus^54^, will be made publicly available at https://github.com/ChristianBergler.
