# Supplementary material for: ANIMAL-SPOT enables animal-independent signal detection and classification using deep learning
Source: Sci Rep. 2022 Dec 19;12:21966. doi: 10.1038/s41598-022-26429-y (PMC9763499; doi:10.1038/s41598-022-26429-y)
Supplement: Supplementary file 1 — Supplementary Information. [file 41598_2022_26429_MOESM1_ESM.pdf]

ANIMAL-SPOT Enables Animal-Independent Signal Detection and Classification Using Deep Learning

Christian Bergler[1,*], Simeon Q Smeele[2,3,4], Stephen A Tyndel[2,5], Alexander Barnhill[1], Sara T Ortiz[6], Ammie K Kalan[7], Rachael Xi Cheng[8], Signe Brinkløv[9], Anna N Osiecka[10], Jakob Tougaard[11], Freja Jakobsen[12], Magnus Wahlberg[12], Elmar Nöth[1], Andreas Maier[1], and Barbara C Klump[2,*]

[1]Friedrich-Alexander-Universität Erlangen-Nürnberg, Department of Computer Science, Pattern Recognition Lab, Erlangen, 91058, Germany
[2]Max Planck Institute of Animal Behavior, Cognitive and Cultural Ecology Lab, Radolfzell, 78315, Germany
[3]Max Planck Institute for Evolutionary Anthropology, Department of Human Behavior, Ecology and Culture, Leipzig, 04103, Germany
[4]University of Konstanz, Biology Department, Konstanz, 78464, Germany
[5]University of Illinois Urbana-Champaign, Department of Natural Resources and Environmental Sciences, Champaign, IL, United States
[6]Max Planck Institute for Biological Intelligence, in foundation, Seewiesen Eberhard-Gwinner-Strasse, 82319 Starnberg, Germany
[7]University of Victoria, Department of Anthropology, Victoria, BC, V8P 5C2, Canada
[8]Leibniz Institute for Zoo and Wildlife Research, Alfred-Kowalke-Straße 17, Berlin, 10315, Germany
[9]Aarhus University, Department of Bioscience, Wildlife Ecology, Rønde, 8410, Denmark
[10]University of Gdańsk, Faculty of Biology, Department of Vertebrate Ecology and Zoology, Gdańsk, 80-308, Poland
[11]Aarhus University, Department of Bioscience, Marine Mammal Research, Roskilde, 4000, Denmark
[12]University of Southern Denmark, Department of Biology, Odense, 5230, Denmark
[*]corresponding authors: Christian Bergler (christian.bergler@fau.de), Barbara Klump (bklump@ab.mpg.de)

Supplementary Information

Animal / Setup	**cockatiel**	**Sulphur-crested cockatoo**	**Peach-fronted conure**	**monk parakeet**	**Blue-/Golden-winged warbler**	**Chinstrap penguin**	**Atlantic cod**	**Harbour seal**	**killer whale[34]**	**Pygmy pipistrelle**	**chimpanzee**
**Microphone/ Hydrophone**	built-in recorder, omni-directional	built-in recorder, omni-directional	free-field microphone Brüel & Kjær, type 4133, pre-amp type 2669	Sennheiser K6/ME67	wildtronics mono-stereo parabolic microphone	Sennheiser ME67	SoundTrap ST300 HF	HTI96min High Tech Inc. Long Beach Mississippi	6 radio-transmitting, various custom-made stationary hydrophones plus custom-made high sensitivity and low noise towed-array	Wildlife Acoustics U1 and U2	Sennheiser directional K6/ME66
**Filtering**	—	—	amplifying 20 dB without filter	—	—	—	—	—	—	16kHz high pass filter or unfiltered (plus inbuilt anti-alias filter in SMs)	—
**Wind Protector/ Extra Gear**	—	—	windscreen UA0237	Rycote Modular Windshield WS 7 Kit	—	MZW 67-PRO velour windshield	—	—	—	U1 supplied and used with foamcap	foam windscreen
**Recorder**	Edic-mini Tiny+ A77	Songmeter SM4 Acoustic recorder (Wildlife Acoustics)	laptop	Sony PCM D100, Marrantz PMD661 MK3, Sony PCM M10	Sony PCM-M10	Olympus LS-100	SoundTrap ST300 HF	DSG-ST (Loggerhead Instruments, Sarasota, USA)	PamGuard, MOTU 24AI and Sony Professional, Walkman WM-D6C or Sony TCD-D3	Songmeters (SM4 and SM4Bat FS) Acoustic recorder (Wildlife Acoustics)	Marantz PMD661 recorder
**Sampling Rate**	16 kHz and 22.05 kHz	48 kHz	44.1 kHz	44.1 kHz	48 kHz	48 kHz	96 kHz	288 kHz	44.1 kHz and 96 kHz	256 kHz	44.1 kHz
**Bit Depth**	16 bit	16 bit	16 bit	16 bit	16 bit	16 bit	16 bit	16 bit	16 bit and 24 bit	16 bit	16 bit
**Location(s)**	Möggingen, Germany	Stanwell Park, Australia	University of Southern Denmark, Denmark	Barcelona, Spain<br>Madrid, Spain<br>Athens, Greece<br>Brussels, Belgium<br>Verona, Italy<br>Bergamo, Italy<br>Lagnago, Italy<br>Pavia, Italy	Wisconsin, USA	Antarctica	Pistilfjordur, Iceland	Limfjord, Denmark	Vancouver Island, Canada	University of Southern Denmark, Denmark<br>Svanninge Bjerge, Denmark<br>Aarhus University Park, Denmark<br>Mols Bjerge National Park, Denmark	Taï National Park, Côte d'Ivoire
**Recording Type**	passive acoustic monitoring	passive acoustic monitoring	focal follow	focal follow, group follow	opportunistic, focal follow	group follow	passive acoustic monitoring	passive acoustic monitoring	passive acoustic monitoring, and group follow	passive acoustic monitoring	focal follow
**Recording Schedule**	3 hours each morning, starting at sunrise	29 min on, 1 min off	during day	during day, 5-20 minute intervals per group/individual	morning and night	during day	24 recording period, recording time selected randomly	continuous	during day, continuous 24/7 concerning the stationary hydrophones, every day if animals were present	sunset to sunrise	during day whenever focal vocalized
**Data Range**	05-2018 until 10-2018	09-2019 until 11-2019	11-2016 until 03-2017	11-2019	04-2016 until 06-2016<br>04-2017 until 06-2017<br>04-2018 until 06-2018	11-2018 until 03-2019	03-2020 until 04-2020	06-2017 until 07-2017	1985-2010 (passive), 2017/2018 (active)	08-2018 until 10-2018<br>04-2019	07-2011 until 05-2012
**Habitat Type**	aviary	residential area/edge of bushland	indoors	urban parks	early successional shrubland	dessertic	fjord, shallow water, bottom partly kelp/sand, swell	shallow fjord waters with sandy bottom	coastal areas around Vancouver Island	vegetation edge, urban and in nature reserves	tropical rain forest
**Background Species (next to pure environmental background noise)**	European blackbird, zebra finch, starling	little corella, catbird, kookaburra, Crimson rosella, king parrot, various songbirds, crickets	—	Ringnecked parakeet, starling, robin, blackbird, human voice	White-throated, sparrow, Black and White warbler, chestnut-sided warbler, grey catbird, indigo bunting	skuas, other penguin species, and human voice	dolphins, humpbacks, other fish species	other marine species	other marine species	other bat species	Diana monkey, King colobus, Western red colobus, bird species, insects species

Supplementary Table 1. Animal-specific recording and data collection setup

Setup \ Scenario		Target versus Noise Detection												Species/Call Type Classification		
**Network Training Parameter**	**Unit**	**CT[1]**	**SC[2]**	**PC[3]**	**MP[4]**	**BWWA[5] GWWA[6]**	**CP[7]**	**AC[8]**	**HS[9]**	**KW[10]**	**PP[11]**	**CI[12]**	**BV[13]**	**BWWA[5] versus GWWA[6]**	**MP[4] call type**	**ComParE PRS[14]**
**Sample Rate**	[kHz]	44.10	44.10	44.10	44.10	44.10	44.10	4.00	44.10	44.10	256.00	44.10	44.10	44.10	44.10	44.10
**Sequence Length**	[ms]	500	500	500	500	640	500	320	1,000	1,280	4	640	450	640	250	400
**FFT-Win**	[samples]	2,048	1,024	1,024	1,024	512	1,024	512	4,096	4,096	256	2,048	1,024	512	512	2,048
**FFT-Hop**	[samples]	172	172	172	172	220	172	10	344	441	8	220	155	220	86	137
**Batch-Size**	[—]	8	8	8	8	8	8	8	8	32	8	8	8	8	8	8
**0/1-dB Normalize**	ref [dB]	20	20	0	20	20	20	0	0	20	0	20	—	20	20	—
	min [dB]	-100	-100	-100	-100	-100	-100	-100	-100	-100	-100	-100	—	-100	-100	—
**Min/Max-Norm**	[—]	—	—	—	—	—	—	—	—	—	—	—	X	—	—	X
**Frequency Range**	min [kHz]	0.00	0.50	0.50	0.50	3.00	0.50	0.03	0.10	0.50	40.00	0.00	0.50	3.00	0.50	0.01
	max [kHz]	8.00	10.00	10.00	10.00	9.00	9.00	0.55	1.30	10.00	100.00	1.50	10.00	9.00	10.00	4.00
**Net Input Size**	F-Bins [–]	256	256	256	256	256	256	256	256	256	256	256	256	256	256	256
	T-Frames [–]	128	128	128	128	128	128	128	128	128	128	128	128	128	128	128
**Learning Rate**	[—]	10^{-4}	10^{-4}	10^{-4}	10^{-4}	10^{-4}	10^{-4}	10^{-4}	10^{-4}	10^{-4}	10^{-4}	10^{-4}	$3.767 * 10^{-3}$	10^{-4}	10^{-4}	10^{-4}
**Max. Training**	[Epoch]	120	120	120	120	120	120	120	120	120	120	120	120	120	120	240
**Early Stopping**	[Epoch]	10	10	10	10	10	10	15	15	10	10	15	34	10	10	10
**Augmentation**	Intensity [dB]	[-6,3]	[-6,3]	[-6,3]	[-6,3]	[-6,3]	[-6,3]	[-6,3]	[-6,3]	[-6,3]	[-6,3]	[-6,3]	[-6,3]	[-6,3]	[-6,3]	[-6,3]
	Pitch [–]	[0.8,1.3]	[0.5,1.5]	[0.5,1.5]	[0.5,1.5]	[0.9,1.2]	[0.5,1.5]	[0.9,1.2]	[0.9,1.1]	[0.5,1.5]	[0.9,1.1]	[0.5,1.5]	[0.75,1.5]	[0.5,1.5]	[0.5,1.5]	[0.9,1.1]
	Time [–]	[0.9,1.4]	[0.5,2.0]	[0.5,2.0]	[0.5,2.0]	[0.7,1.2]	[0.5,2.0]	[0.9,1.5]	[1.1,1.5]	[0.5,2.0]	[0.9,1.3]	[0.5,2.0]	[0.9,1.1]	[0.5,2.0]	[1.4,2.0]	[1.2,1.5]
	Noise [dB]	[-3,12]	[—]	[—]	[-3,12]	[-3,12]	[—]	[—]	[-3,12]	[-3,12]	[-3,12]	[-3,12]	[8,12]	[—]	[—]	[—]

[1] cockatiel (CT) [2] Sulphur-crested cockatoo (SC) [3] Peach-fronted conure (PC) [4] monk parakeet (MP)
[5] Blue-winged warbler (BWWA), [6] Golden-winged warbler (GWWA) [7] Chinstrap penguin (CP) [8] Atlantic cod (AC) [9] Harbour seal (HS) [10] killer whale (KW)[34]
[11] Pygmy pipistrelle (PP) [12] chimpanzee (CI) [13] BirdVox (BV) [14] Computational Paralinguistics Challenge Primate (ComParE PRS)[37,38]

Supplementary Table 2. Network-Hyperparameters of ANIMAL-SPOT for each animal database (see Table 1, 2, 3, and 4)

split / dataset		training				validation				test			
		samples				samples				samples			
		target	noise	∑	%	target	noise	∑	%	target	noise	∑	%
**cockatiel**	**3,111**	890	1,253	2,143	68.88	188	393	581	18.68	193	194	387	12.44
**Sulphur-crested cockatoo**	**3,640**	1,040	1,476	2,516	69.12	225	367	592	16.26	230	302	532	14.62
**Peach-fronted conure**	**2,126**	822	667	1,489	70.04	176	143	319	15.00	176	142	318	14.96
**monk parakeet**	**6,745**	2,168	2,407	4,575	67.83	340	563	903	13.39	625	642	1,267	18.78
**Blue-/Golden-winged warbler**	**5,047**	1,130	2,401	3,531	69.96	242	515	757	15.00	244	515	759	15.04
**Chinstrap penguin**	**4,360**	675	2,400	3,075	70.52	106	618	724	16.61	125	436	561	12.87
**Atlantic cod**	**1,249**	267	606	873	69.90	57	130	187	14.97	58	131	189	15.13
**Harbour seal**	**5,145**	2,028	1,312	3,340	64.92	437	385	822	15.98	435	548	983	19.10
**killer whale**[34]	**61,427**	11,509	34,848	46,357	75.47	2,942	4,681	7,623	12.41	2,653	4,794	7,447	12.12
**Pygmy pipistrelle**	**5,060**	1,095	2,355	3,450	68.18	238	796	1,034	20.43	237	339	576	11.38
**chimpanzee**	**12,384**	4,955	3,714	8,669	70.00	1,062	796	1,858	15.00	1,062	795	1,857	15.00

Supplementary Table 3. Binary (target vs. noise) detection – training, validation, and test data distributions

split / dataset		training						validation						test					
		samples						samples						samples					
		b[1]	g[2]	o[3]	n[4]	∑	%	b[1]	g[2]	o[3]	n[4]	∑	%	b[1]	g[2]	o[3]	n[4]	∑	%
**Warbler species**	**3,158**	495	636	379	700	2,210	70.0	106	137	82	150	475	15.0	106	136	81	150	473	15.0

[1] Blue-winged warbler (b) [2] Golden-winged warbler (g) [3] other bird (o) [4] noise (n)

Supplementary Table 4. Multi-class Blue-winged and Golden-winged warbler identification – training, validation, test data distribution

split / dataset		training						validation						test					
		samples						samples						samples					
		c[1]	a[2]	o[3]	n[4]	∑	%	c[1]	a[2]	o[3]	n[4]	∑	%	c[1]	a[2]	o[3]	n[4]	∑	%
**monk parakeet call types**	**3,251**	480	559	535	700	2,274	70.0	105	119	115	159	498	15.3	104	120	114	141	479	14.7

[1] contact call (c) [2] alarm call (a) [3] other call (o) [4] noise (n)

Supplementary Table 5. Multi-class monk parakeet call type identification – training, validation, and test data distribution

dataset \ split		training							validation							test						
		samples							samples							samples						
		c[1]	m[2]	r[3]	g[4]	n[5]	∑	%	c[1]	m[2]	r[3]	g[4]	n[5]	∑	%	c[1]	m[2]	r[3]	g[4]	n[5]	∑	%
**Primate species**	**34,589**	4,434	1748	417	317	6,917	13,833	40.0	4,434	1748	417	317	6,917	13,833	40.0	2,218	875	210	159	3,461	6,923	20.0

[1] chimpanzee (c) [2] mandrills (m) [3] Red-capped mangabeys (r) [4] guenons (g) [5] noise (n)

Supplementary Table 6. Multi-class ComParE-PRS[37,38] primate species identification – training, validation, test data distribution (train equal validation)

	ANIMAL-SPOT Results																	Unseen Recording Results (ANIMAL-SPOT-S)											
	E[12]	ACC[13]			TPR[14]			FPR[15]			PREC[16]			F1[17]			AUC	AUC[18]				PREC ($\delta \geq$ 0.9)[19]				TPR ($\delta \geq$ 0.9)			
	Best	Tr[20]	Val[21]	Test[22]	Tr	Val	Test	Tr	Val	Test	Tr	Val	Test	Tr	Val	Test	Test	A[23]	B[23]	C[23]	∑	A	B	C	∑	A	B	C	∑
**CT[1]**	42	0.990	0.986	0.997	0.983	0.989	0.995	0.006	0.015	0.000	0.992	0.969	1.000	0.988	0.979	0.997	1.000	0.897	0.953	0.914	0.917	0.958	0.974	1.000	0.969	0.327	0.563	0.000	0.433
**SC[2]**	66	0.974	0.958	0.953	0.968	0.942	0.922	0.022	0.033	0.023	0.968	0.946	0.968	0.968	0.944	0.944	0.988	0.892	0.915	0.995	0.991	0.963	0.841	1.000	0.940	0.964	0.646	0.513	0.888
**PC[3]**	54	0.989	0.994	0.994	0.985	0.989	0.989	0.006	0.000	0.000	0.995	1.000	1.000	0.990	0.994	0.994	0.999	0.971	0.999	0.998	0.987	0.947	0.931	0.933	0.946	0.989	1.000	1.000	0.990
**MP[4]**	36	0.979	0.991	0.994	0.977	0.988	0.994	0.020	0.007	0.006	0.978	0.988	0.994	0.978	0.988	0.994	1.000	0.960	0.975	0.986	0.975	0.975	0.963	0.996	0.972	0.662	0.677	0.812	0.681
**WA[5]**	68	0.993	1.000	0.992	0.985	1.000	1.000	0.003	0.000	0.012	0.993	1.000	0.976	0.989	1.000	0.988	1.000	0.946	0.939	0.961	0.950	0.935	0.948	0.977	0.953	0.862	0.845	0.774	0.826
**CP[6]**	68	0.984	0.978	0.973	0.960	0.896	0.968	0.010	0.008	0.025	0.966	0.950	0.917	0.963	0.922	0.942	0.996	0.976	0.954	0.931	0.952	1.000	1.000	0.981	0.995	0.769	0.474	0.730	0.728
**AC[7]**	18	0.983	0.995	0.989	0.966	1.000	0.966	0.010	0.008	0.000	0.977	0.983	1.000	0.972	0.991	0.982	0.998	0.991	0.996	0.906	0.976	0.995	0.998	1.000	0.996	0.698	0.751	0.000	0.651
**HS[8]**	24	0.974	0.981	0.982	0.982	0.993	0.995	0.039	0.034	0.029	0.975	0.971	0.964	0.979	0.982	0.980	0.997	0.936	0.898	0.936	0.927	0.924	1.000	0.966	0.955	0.668	0.563	0.730	0.647
**KW[9]**	26	0.957	0.947	0.950	0.881	0.908	0.938	0.017	0.028	0.044	0.944	0.954	0.923	0.911	0.931	0.930	0.983	0.926	0.915	0.999	0.960	0.932	0.927	0.895	0.931	0.899	0.608	1.000	0.860
**PP[10]**	26	0.986	0.985	0.998	0.980	1.000	1.000	0.011	0.020	0.003	0.975	0.937	0.996	0.978	0.967	0.998	1.000	0.979	0.985	0.975	0.971	0.996	0.926	0.950	0.960	0.809	0.845	0.821	0.822
**CI[11]**	30	0.966	0.938	0.945	0.985	0.961	0.951	0.060	0.094	0.063	0.957	0.932	0.953	0.971	0.946	0.952	0.992	0.884	0.993	0.974	0.944	1.000	1.000	1.000	1.000	0.063	0.551	0.069	0.277
**ø**	42	0.980	0.978	0.979	0.968	0.970	0.974	0.019	0.022	0.019	0.975	0.966	0.972	0.972	0.968	0.973	0.996	0.942	0.957	0.961	0.959	0.966	0.955	0.973	0.965	0.701	0.684	0.586	0.709

[1] CT = cockatiel [2] SC = Sulphur-crested cockatoo [3] PC = Peach-fronted conure [4] MP = monk parakeet [5] WA = Blue-/Golden-winged warbler
[6] CP = Chinstrap penguin [7] AC = Atlantic cod [8] HS = Harbour seal [9] KW = killer whale[34] [10] PP = Pygmy pipistrelle [11] CI = chimpanzee [12] E = Epoch
[13] ACC = Accuracy [14] TPR = True Positive Rate [15] FPR = False Positive Rate [16] PREC = Precision [17] F1 = F1-Score [18] AUC = area under the ROC curve
[19] $\delta \geq 0.9$ = Network prediction confidence threshold δ larger 90 % [20] Tr = Training [21] Val = Validation [22] Test = Testing [23] A = Unseen Recording A, B, and C

Supplementary Table 7. Summarized target/noise detection results for all 10 distinct animal species and 1 additional genus, according to Supplementary Figure S2 – S12

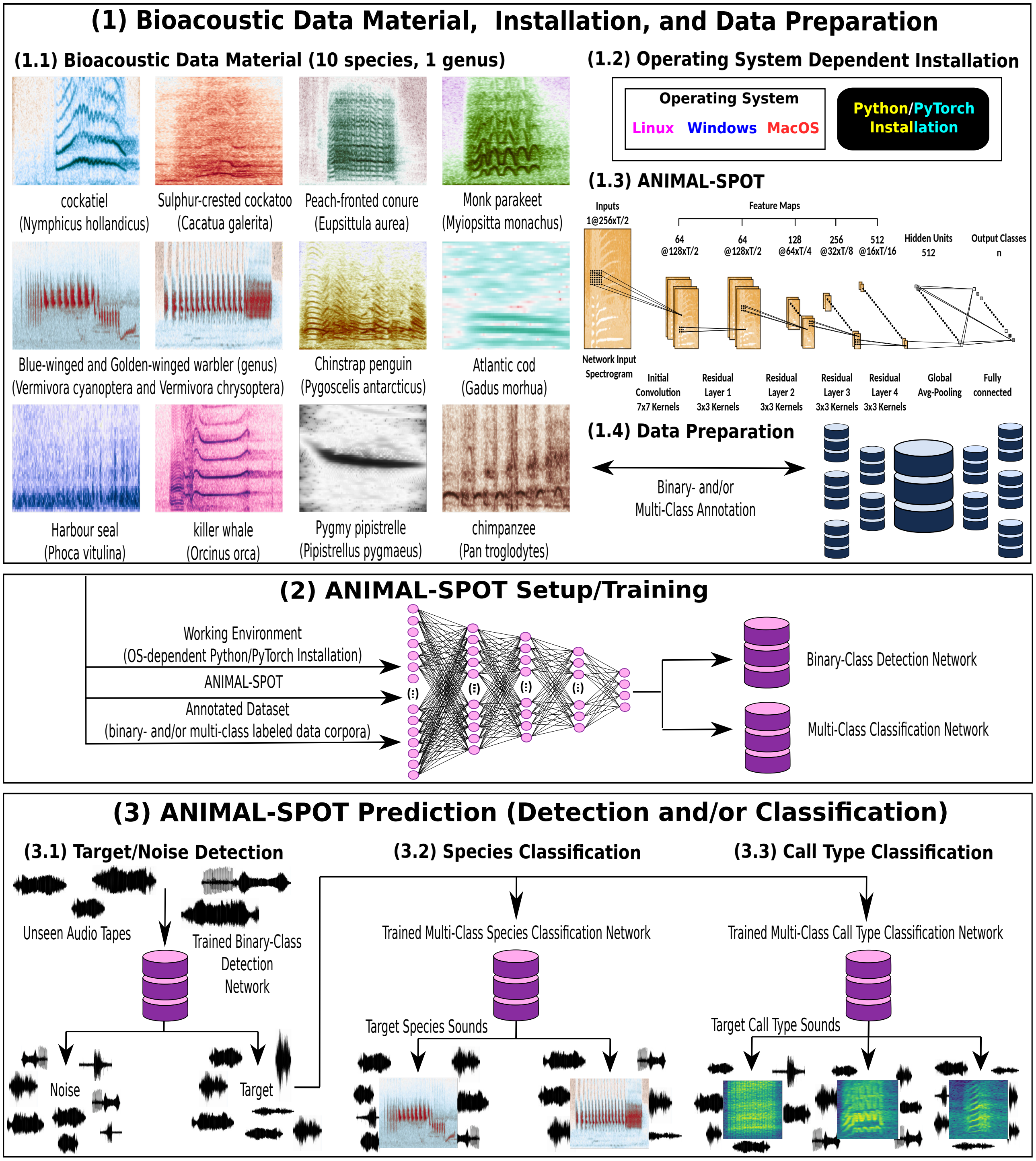

Supplementary Figure 1. Visualization of the entire ANIMAL-SPOT concept, structured in three sequentially-ordered stages, starting with: (1) bioacoustic data material, installation, and data preparation, sub-divided into (1.1) bioacoustic data corpora utilized within this study, (1.2) operating-system-dependent installations, (1.3) network architecture and corresponding source code, and (1.4) data preparation, followed by (2) animal-/and scenario-specific network setups and training procedures, completed with (3) ANIMAL-SPOT prediction with respect to detection and/or classification, further structured in (3.1) target/noise detection, (3.2) species classification, and (3.3) call type identification, all together summarized within a publicly available user guide[33], enabling researchers to build task- and animal-specific deep bioacoustic models (created via Inkscape[39], Version 0.92.3).

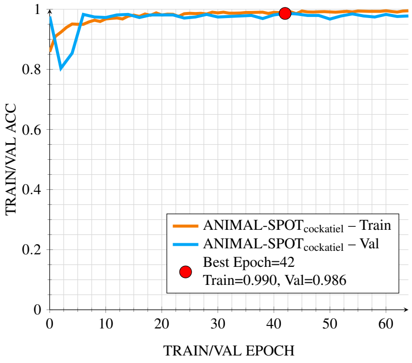

(1.1) Accuracy (ACC)

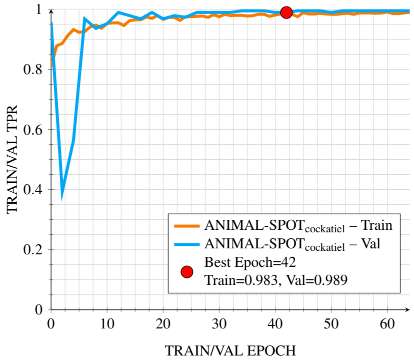

(1.2) True-Positive-Rate (TPR)

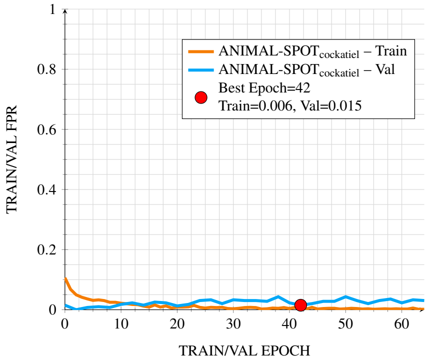

(1.3) False-Positive-Rate (FPR)

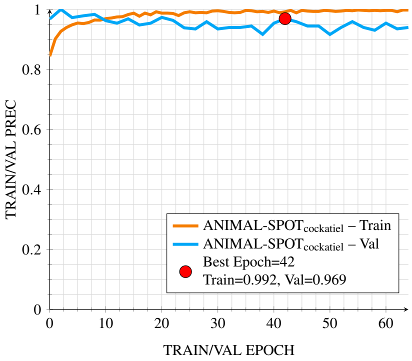

(1.4) Precision (PREC)

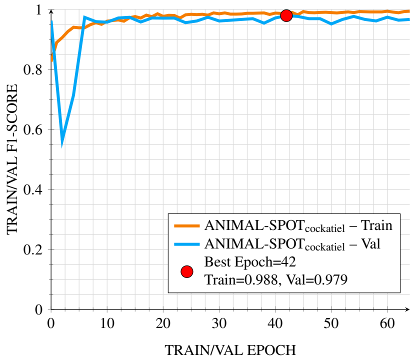

(1.5) F1-Score

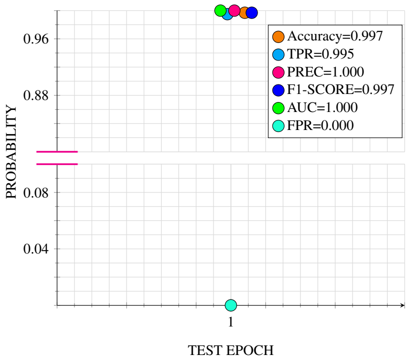

(1.6) Test Metrics

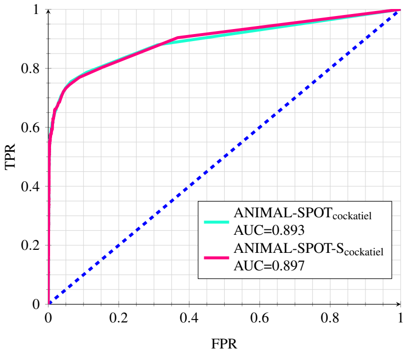

(1.7) ROC-Curve Recording A (high)
Duration [s] = 3,639.57
Target [s] = 76.35, Noise [s] = 3,563.22
Predicted Frames = 36,389

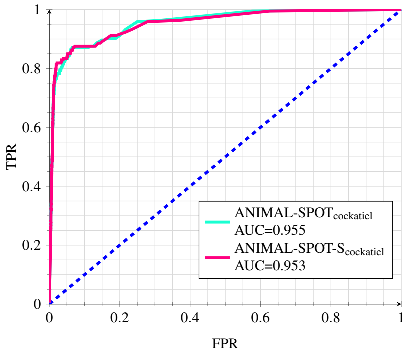

(1.8) ROC-Curve Recording B (med)
Duration [s] = 3,627.13
Target [s] = 68.86, Noise [s] = 3,558.27
Predicted Frames = 36,265

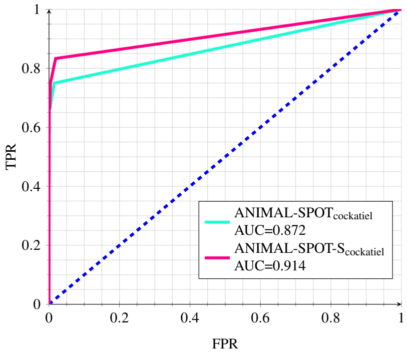

(1.9) ROC-Curve Recording C (low)
Duration [s] = 3,627.13
Target [s] = 2.50, Noise [s] = 3,624.63
Predicted Frames = 36,265

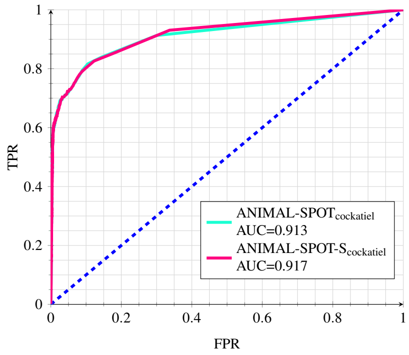

(1.10) Overall ROC-Curve
Duration [s] = 10,893.83
Target [s] = 147.71, Noise [s] = 10,746.12
Predicted Frames = 108,919

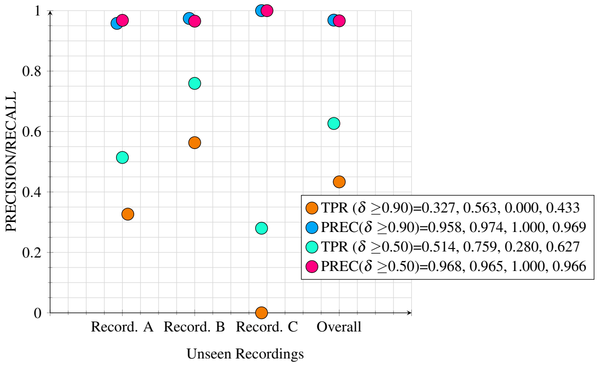

(1.11) ANIMAL-SPOT-S$_{cockatiel}$ – Precision and Recall (TPR)
Threshold ($\delta \geq$0.90 and $\delta \geq$0.50)
Annotation merge factor ξ [s] = 0.35
Prediction overlap factor λ [s] = 0.35

Supplementary Figure 2. cockatiel (*Nymphicus Hollandicus*) – Model metrics, unseen recording-based ROC-Curves and time-wise precision and recall, Network prediction settings: window-length $\varepsilon = 700$ ms, step-size $\kappa = 100$ ms (created via Inkscape[39], Version 0.92.3)

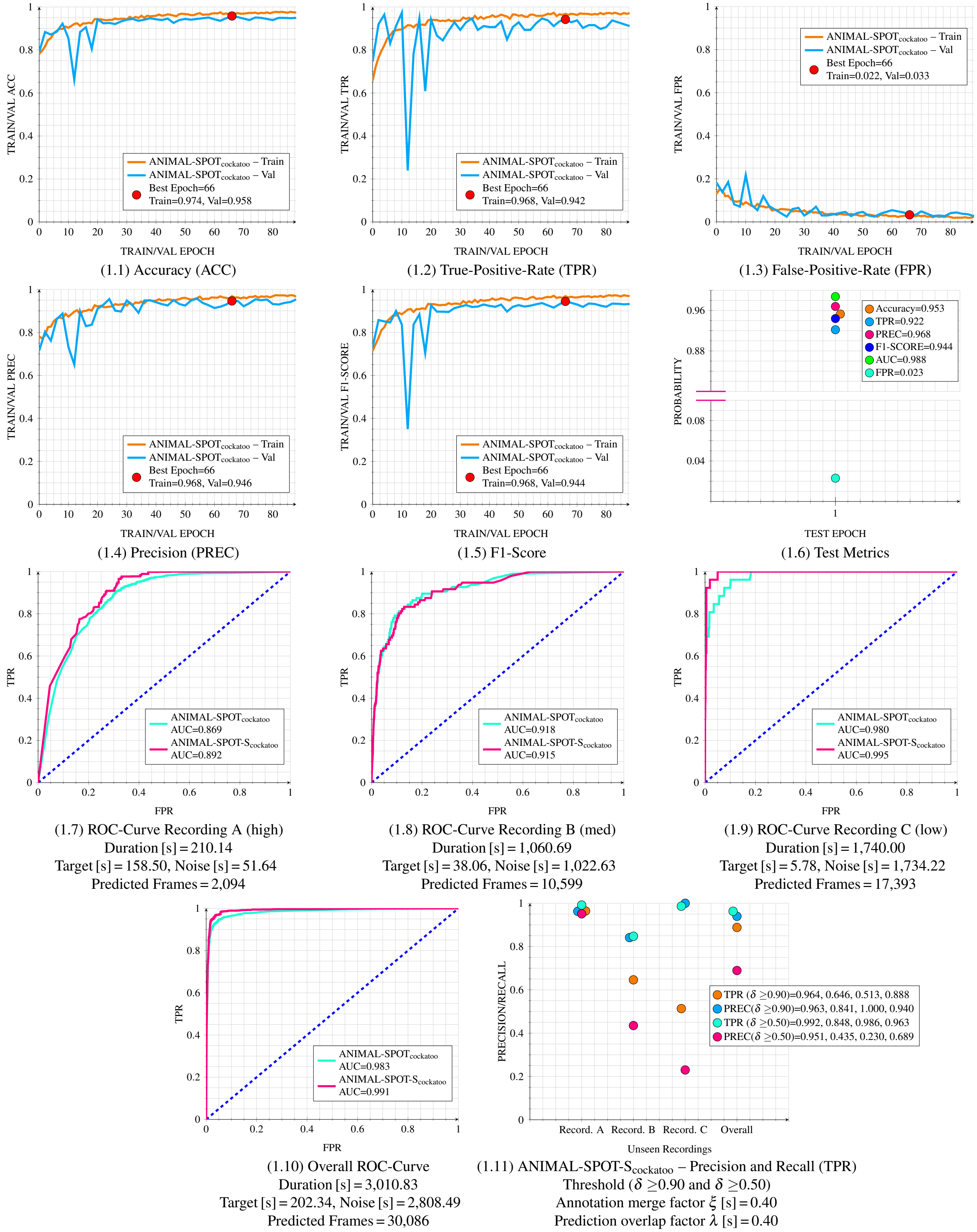

Supplementary Figure 3. Sulfur-crested cockatoo (*Cacatua Galerita*) – Model metrics, unseen recording-based ROC-Curves and time-wise precision and recall, Network prediction settings: window-length $\varepsilon = 800$ ms, step-size $\kappa = 100$ ms (created via Inkscape[39], Version 0.92.3)

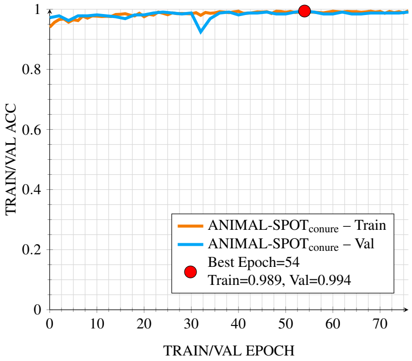

(1.1) Accuracy (ACC)

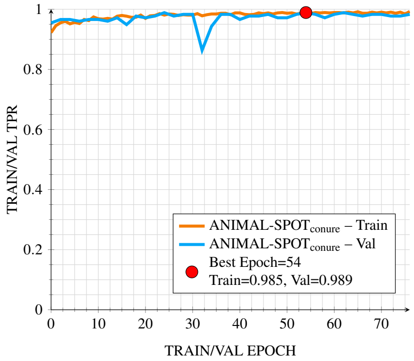

(1.2) True-Positive-Rate (TPR)

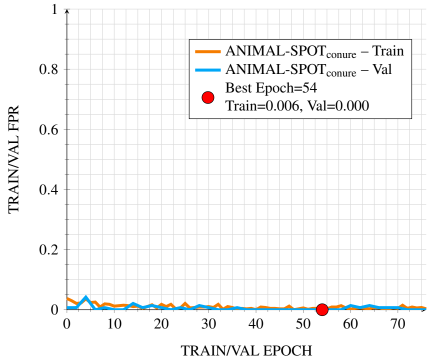

(1.3) False-Positive-Rate (FPR)

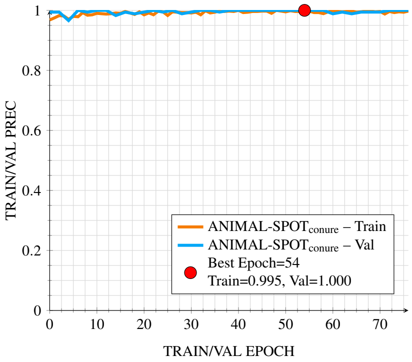

(1.4) Precision (PREC)

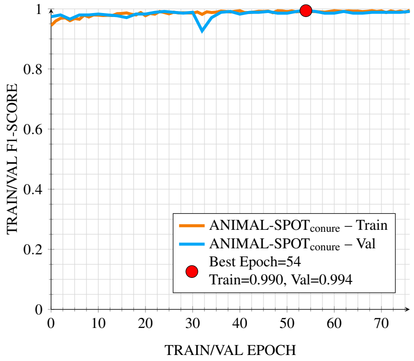

(1.5) F1-Score

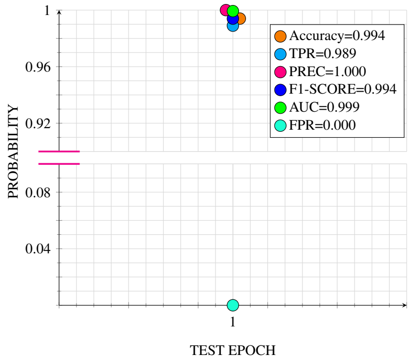

(1.6) Test Metrics

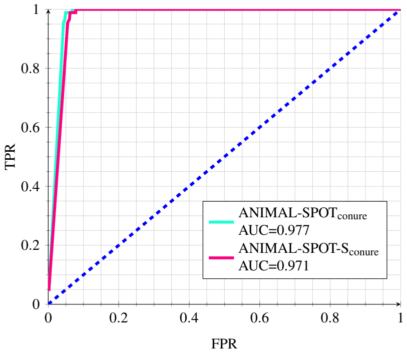

(1.7) ROC-Curve Recording A (high)
Duration [s] = 126.94
Target [s] = 12.48, Noise [s] = 114.46
Predicted Frames = 1,267

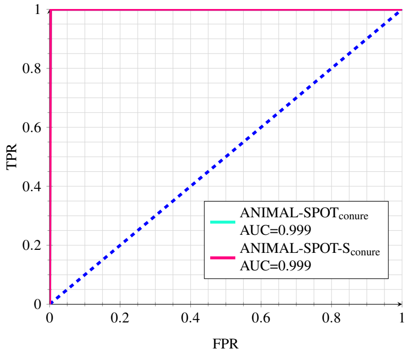

(1.8) ROC-Curve Recording B (med)
Duration [s] = 40.21
Target [s] = 0.29, Noise [s] = 39.92
Predicted Frames = 400

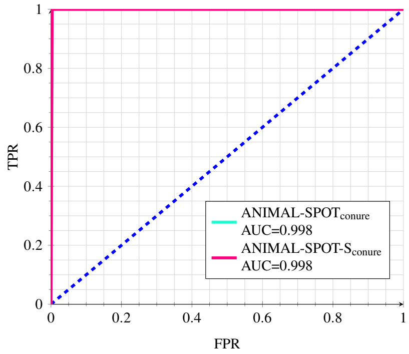

(1.9) ROC-Curve Recording C (low)
Duration [s] = 118.13
Target [s] = 0.53, Noise [s] = 117.60
Predicted Frames = 1,179

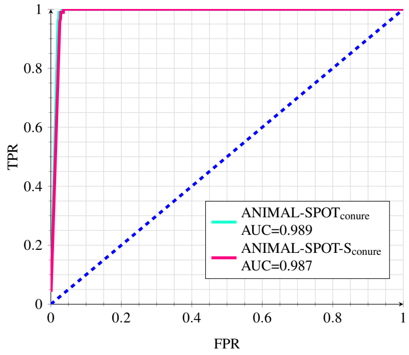

(1.10) Overall ROC-Curve
Duration [s] = 285.28
Target [s] = 13.30, Noise [s] = 271.98
Predicted Frames = 2,846

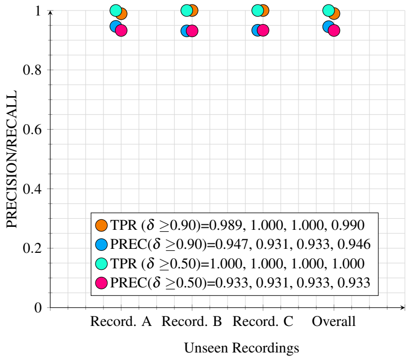

(1.11) ANIMAL-SPOT-S$_{conure}$ – Precision and Recall (TPR)
Threshold ($\delta \geq 0.90$ and $\delta \geq 0.50$)
Annotation merge factor ξ [s] = 0.15
Prediction overlap factor λ [s] = 0.15

Supplementary Figure 4. Peach-fronted conure (*Eupsittula Aurea*) – Model metrics, unseen recording-based ROC-Curves and time-wise precision and recall, Network prediction settings: window-length $\varepsilon = 300$ ms, step-size $\kappa = 100$ ms (created via Inkscape[39], Version 0.92.3)

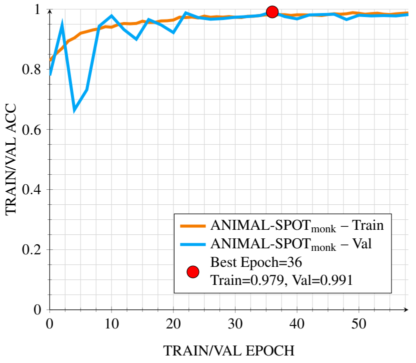

(1.1) Accuracy (ACC)

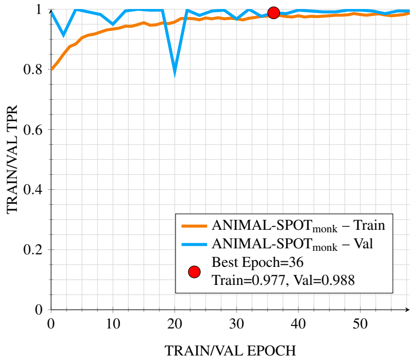

(1.2) True-Positive-Rate (TPR)

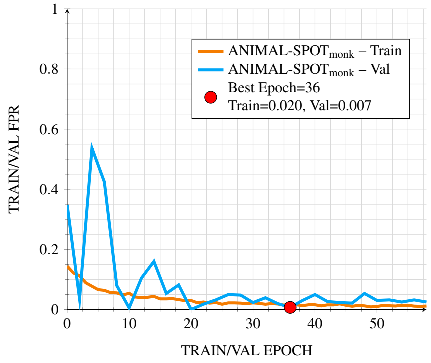

(1.3) False-Positive-Rate (FPR)

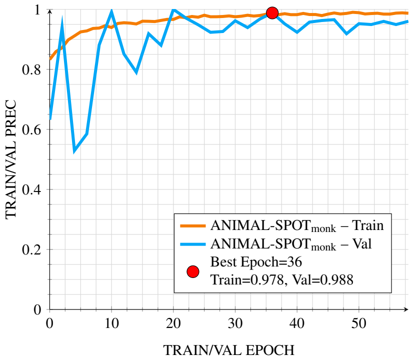

(1.4) Precision (PREC)

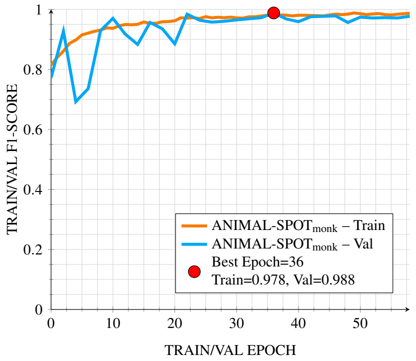

(1.5) F1-Score

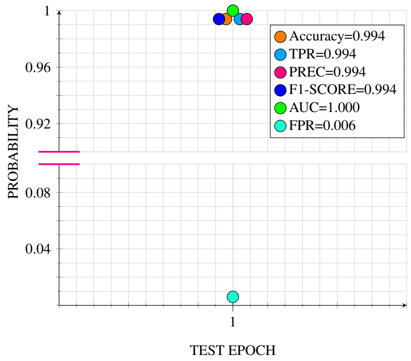

(1.6) Test Metrics

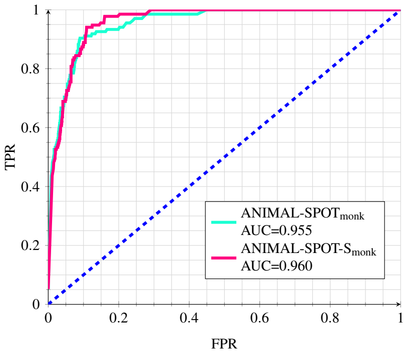

(1.7) ROC-Curve Recording A (high)
Duration [s] = 199.29
Target [s] = 20.64, Noise [s] = 178.65
Predicted Frames = 1,990

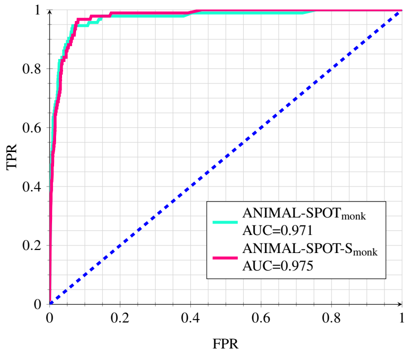

(1.8) ROC-Curve Recording B (med)
Duration [s] = 304.99
Target [s] = 13.91, Noise [s] = 291.08
Predicted Frames = 3,047

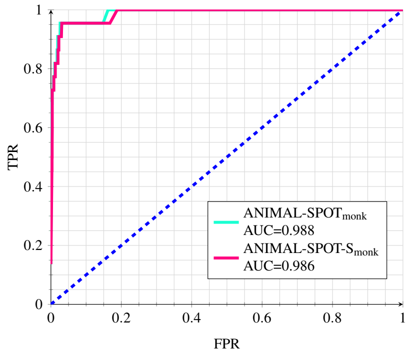

(1.9) ROC-Curve Recording C (low)
Duration [s] = 291.24
Target [s] = 3.37, Noise [s] = 287.87
Predicted Frames = 2,910

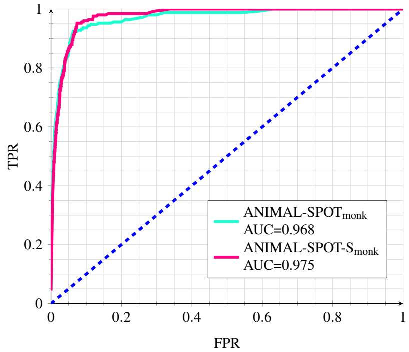

(1.10) Overall ROC-Curve
Duration [s] = 795.52
Target [s] = 37.92, Noise [s] = 757.60
Predicted Frames = 7,947

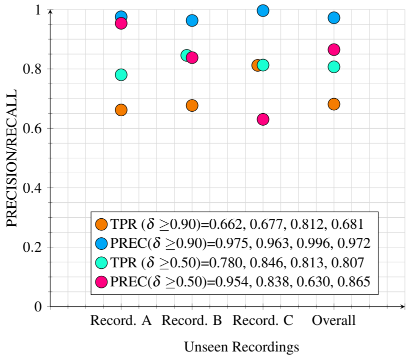

(1.11) ANIMAL-SPOT-S$_{monk}$ – Precision and Recall (TPR)
Threshold ($\delta \geq$0.90 and $\delta \geq$0.50)
Annotation merge factor ξ [s] = 0.15
Prediction overlap factor λ [s] = 0.15

Supplementary Figure 5. monk parakeet (*Myiopsitta Monachus*) – Model metrics, unseen recording-based ROC-Curves and time-wise precision and recall, Network prediction settings: window-length $\varepsilon = 300$ ms, step-size $\kappa = 100$ ms (created via Inkscape[39], Version 0.92.3)

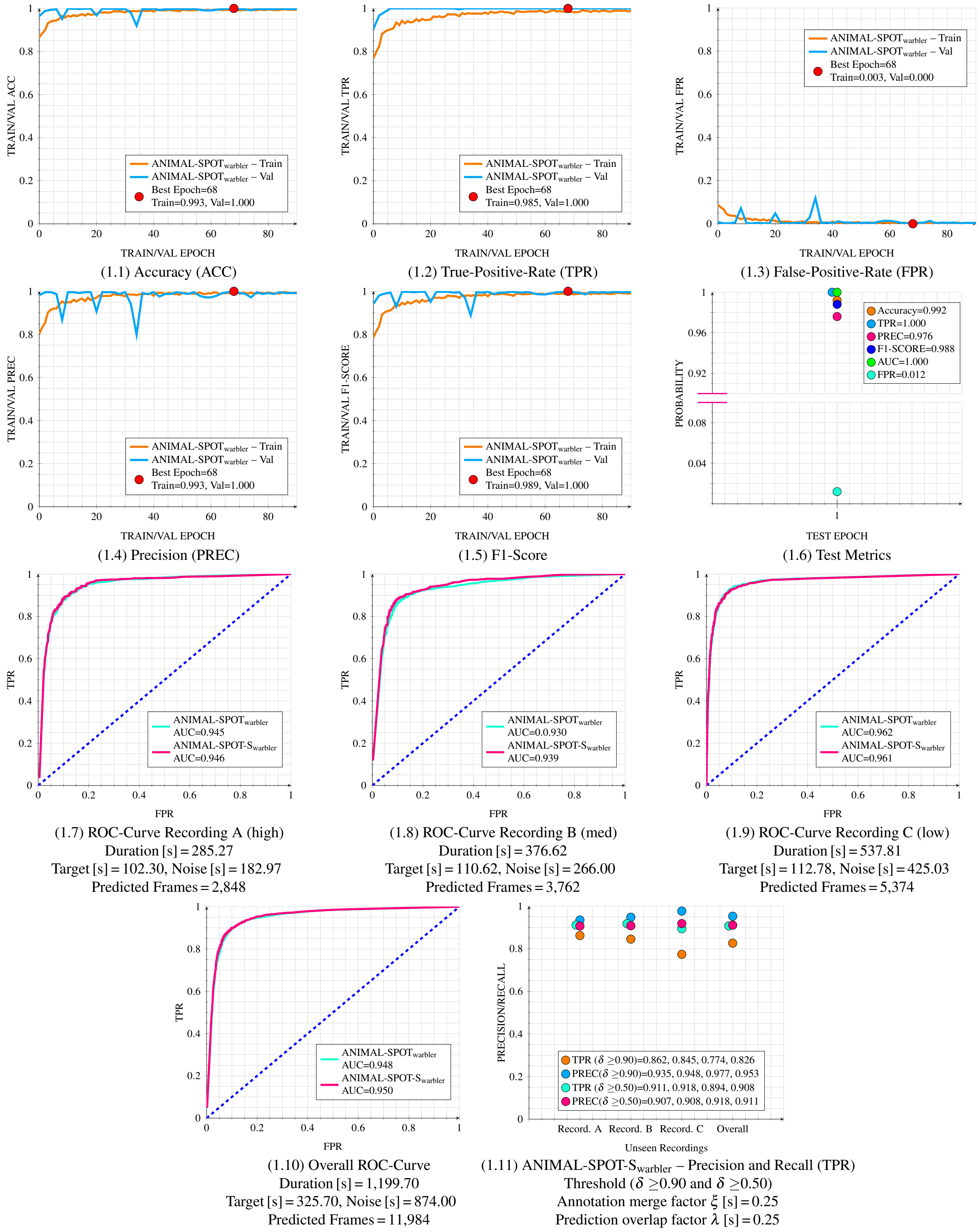

(1.1) Accuracy (ACC)

(1.2) True-Positive-Rate (TPR)

(1.3) False-Positive-Rate (FPR)

(1.4) Precision (PREC)

(1.5) F1-Score

(1.6) Test Metrics

(1.7) ROC-Curve Recording A (high)
Duration [s] = 285.27
Target [s] = 102.30, Noise [s] = 182.97
Predicted Frames = 2,848

(1.8) ROC-Curve Recording B (med)
Duration [s] = 376.62
Target [s] = 110.62, Noise [s] = 266.00
Predicted Frames = 3,762

(1.9) ROC-Curve Recording C (low)
Duration [s] = 537.81
Target [s] = 112.78, Noise [s] = 425.03
Predicted Frames = 5,374

(1.10) Overall ROC-Curve
Duration [s] = 1,199.70
Target [s] = 325.70, Noise [s] = 874.00
Predicted Frames = 11,984

(1.11) ANIMAL-SPOT-$S_{warbler}$ – Precision and Recall (TPR)
Threshold ($\delta \geq 0.90$ and $\delta \geq 0.50$)
Annotation merge factor ξ [s] = 0.25
Prediction overlap factor λ [s] = 0.25

Supplementary Figure 6. Blue-winged warbler (*Vermivora cyanoptera*) and Golden-winged warbler (*Vermivora chrysoptera*) – Model metrics, unseen recording-based ROC-Curves and time-wise precision and recall, Network prediction settings: window-length $\varepsilon = 500$ ms, step-size $\kappa = 100$ ms (created via Inkscape[39], Version 0.92.3)

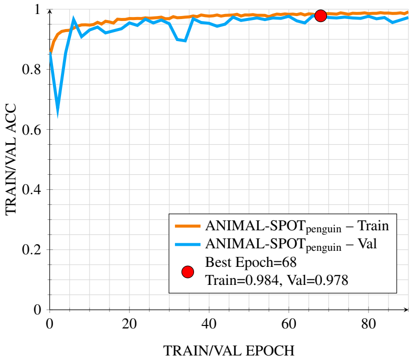

(1.1) Accuracy (ACC)

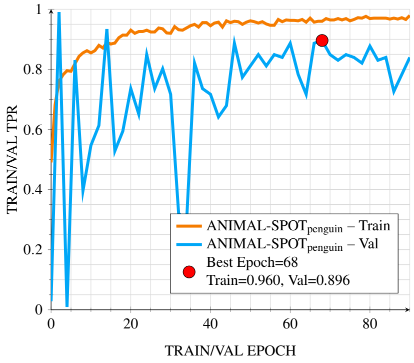

(1.2) True-Positive-Rate (TPR)

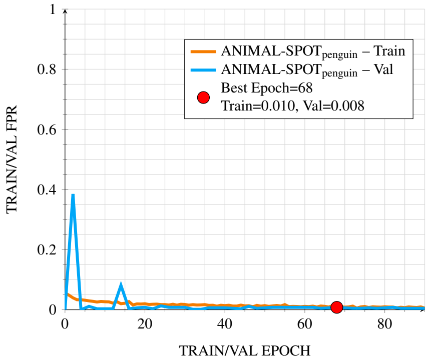

(1.3) False-Positive-Rate (FPR)

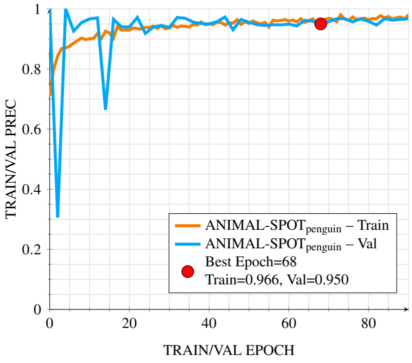

(1.4) Precision (PREC)

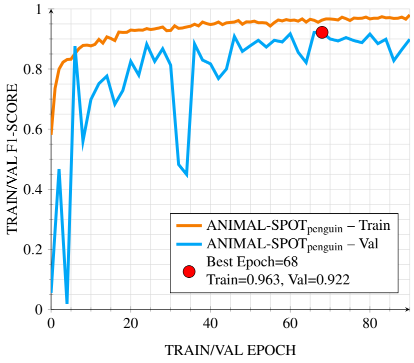

(1.5) F1-Score

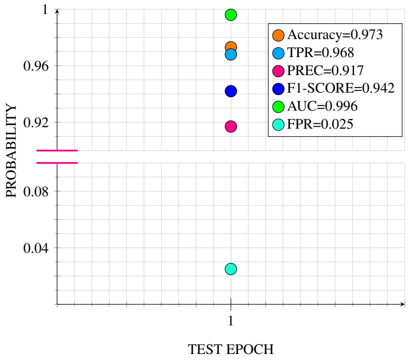

(1.6) Test Metrics

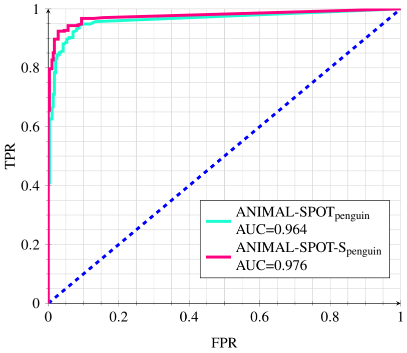

(1.7) ROC-Curve Recording A (high)
Duration [s] = 65.98
Target [s] = 38.46, Noise [s] = 27.52
Predicted Frames = 654

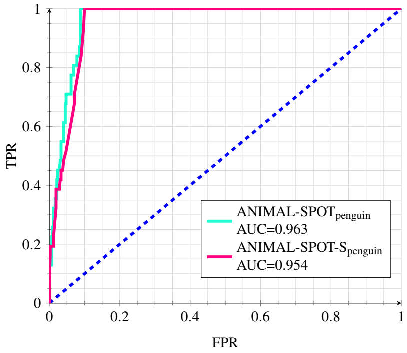

(1.8) ROC-Curve Recording B (med)
Duration [s] = 45.74
Target [s] = 2.86, Noise [s] = 42.88
Predicted Frames = 452

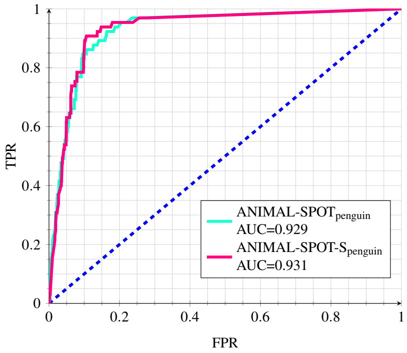

(1.9) ROC-Curve Recording C (low)
Duration [s] = 133.23
Target [s] = 8.15, Noise [s] = 125.08
Predicted Frames = 1,327

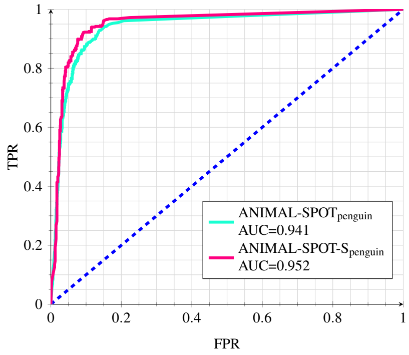

(1.10) Overall ROC-Curve
Duration [s] = 244.95
Target [s] = 49.47, Noise [s] = 195.48
Predicted Frames = 2,433

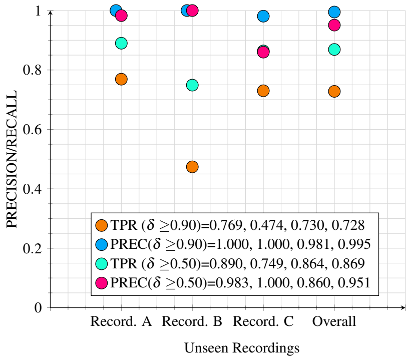

(1.11) ANIMAL-SPOT-S$_{penguin}$ – Precision and Recall (TPR)
Threshold ($\delta \geq$0.90 and $\delta \geq$0.50)
Annotation merge factor ξ [s] = 0.30
Prediction overlap factor λ [s] = 0.30

Supplementary Figure 7. Chinstrap penguin (*Pygoscelis Antarcticus*) – Model metrics, unseen recording-based ROC-Curves and time-wise precision and recall, Network prediction settings: window-length $\varepsilon = 600$ ms, step-size $\kappa = 100$ ms (created via Inkscape[39], Version 0.92.3)

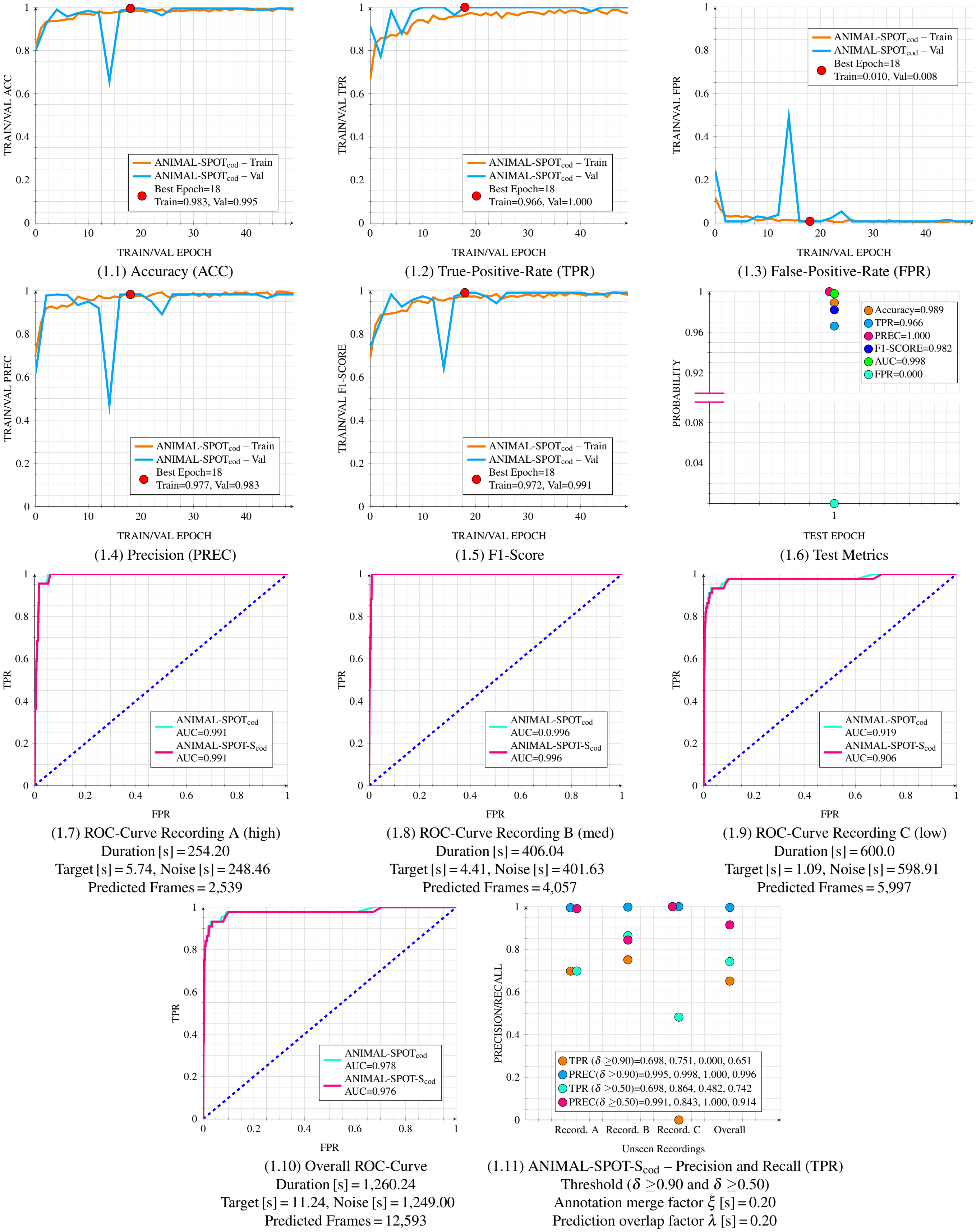

(1.1) Accuracy (ACC)

(1.2) True-Positive-Rate (TPR)

(1.3) False-Positive-Rate (FPR)

(1.4) Precision (PREC)

(1.5) F1-Score

(1.6) Test Metrics

(1.7) ROC-Curve Recording A (high)
Duration [s] = 254.20
Target [s] = 5.74, Noise [s] = 248.46
Predicted Frames = 2,539

(1.8) ROC-Curve Recording B (med)
Duration [s] = 406.04
Target [s] = 4.41, Noise [s] = 401.63
Predicted Frames = 4,057

(1.9) ROC-Curve Recording C (low)
Duration [s] = 600.0
Target [s] = 1.09, Noise [s] = 598.91
Predicted Frames = 5,997

(1.10) Overall ROC-Curve
Duration [s] = 1,260.24
Target [s] = 11.24, Noise [s] = 1,249.00
Predicted Frames = 12,593

(1.11) ANIMAL-SPOT-S_{cod} – Precision and Recall (TPR)
Threshold ($\delta \geq 0.90$ and $\delta \geq 0.50$)
Annotation merge factor ξ [s] = 0.20
Prediction overlap factor λ [s] = 0.20

Supplementary Figure 8. Atlantic cod (*Gadus morhua*) – Model metrics, unseen recording-based ROC-Curves and time-wise precision and recall, Network prediction settings: window-length $\varepsilon = 400$ ms, step-size $\kappa = 100$ ms (created via Inkscape[39], Version 0.92.3)

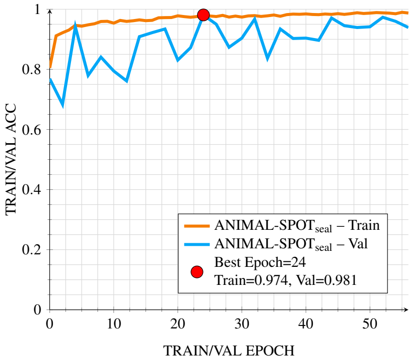

(1.1) Accuracy (ACC)

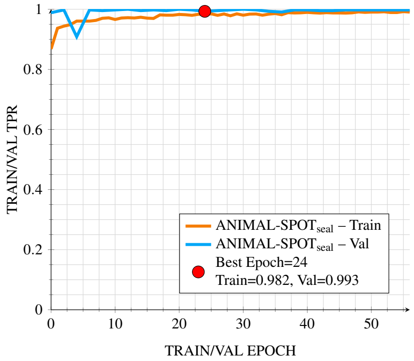

(1.2) True-Positive-Rate (TPR)

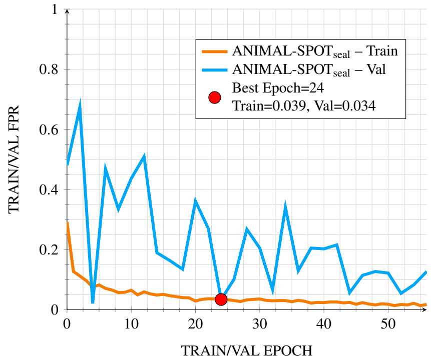

(1.3) False-Positive-Rate (FPR)

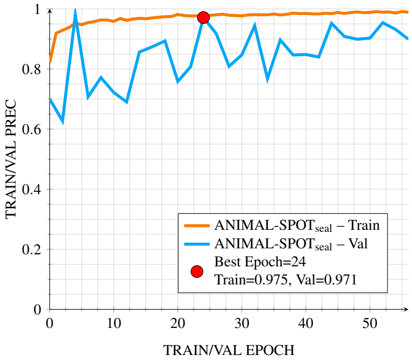

(1.4) Precision (PREC)

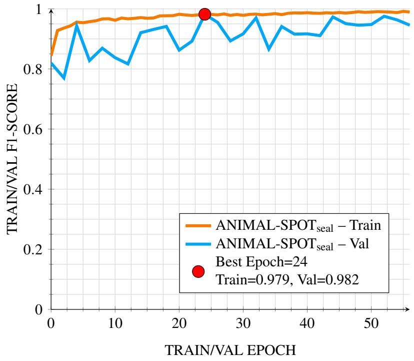

(1.5) F1-Score

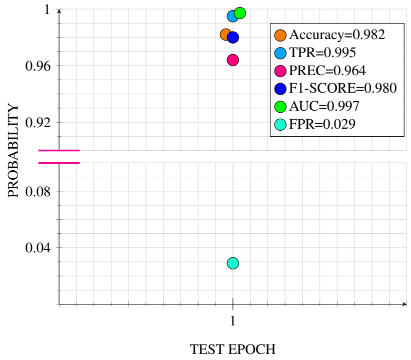

(1.6) Test Metrics

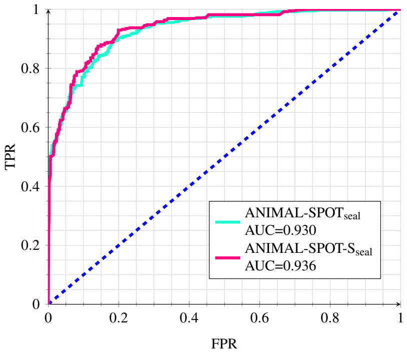

(1.7) ROC-Curve Recording A (high)
Duration [s] = 600.00
Target [s] = 201.93, Noise [s] = 398.07
Predicted Frames = 1,197

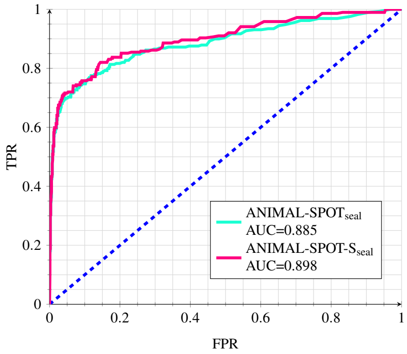

(1.8) ROC-Curve Recording B (med)
Duration [s] = 600.00
Target [s] = 157.65, Noise [s] = 442.35
Predicted Frames = 1,197

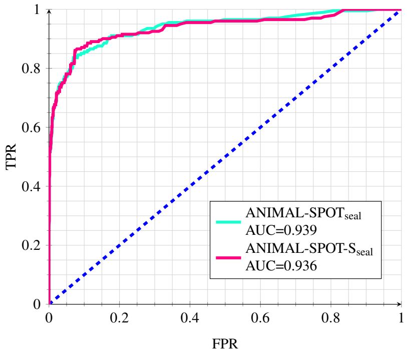

(1.9) ROC-Curve Recording C (low)
Duration [s] = 600.00
Target [s] = 107.80, Noise [s] = 492.20
Predicted Frames = 1,197

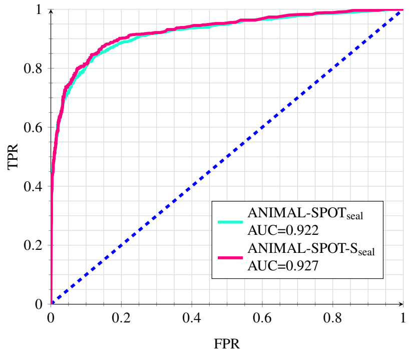

(1.10) Overall ROC-Curve
Duration [s] = 1,800.00
Target [s] = 467.38, Noise [s] = 1,332.62
Predicted Frames = 3,591

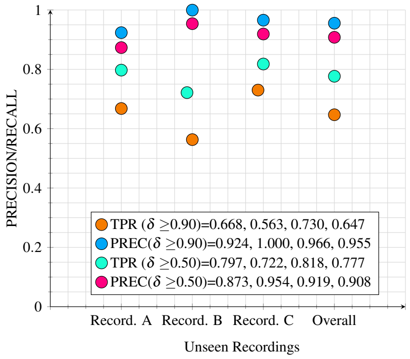

(1.11) ANIMAL-SPOT-S$_{seal}$ – Precision and Recall (TPR)
Threshold ($\delta \geq$0.90 and $\delta \geq$0.50)
Annotation merge factor ξ [s] = 1.00
Prediction overlap factor λ [s] = 1.00

Supplementary Figure 9. Harbour seal (*Phoca Vitulina*) – Model metrics, unseen recording-based ROC-Curves and time-wise precision and recall, Network prediction settings: window-length $\varepsilon = 2000$ ms, step-size $\kappa = 500$ ms (created via Inkscape[39], Version 0.92.3)

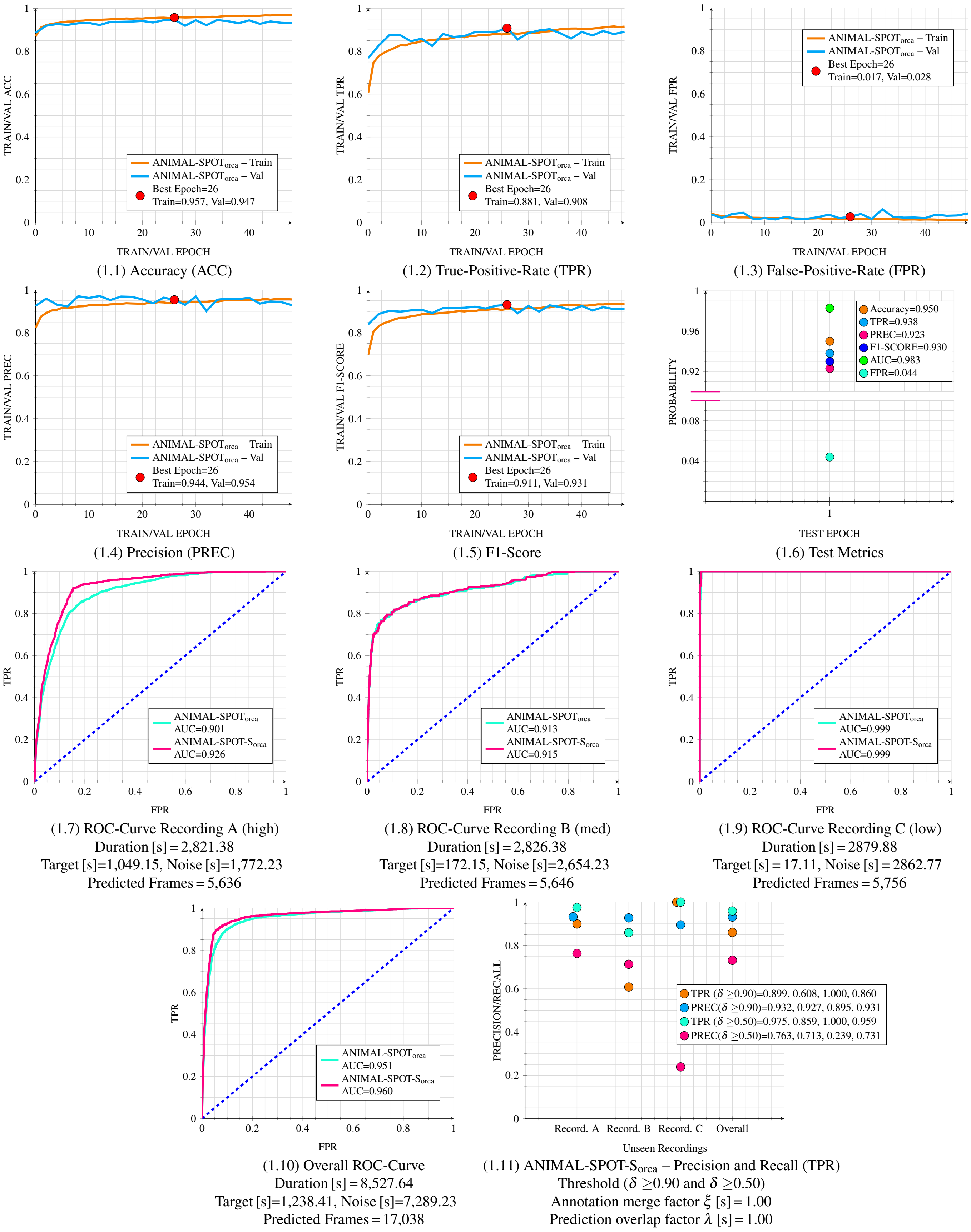

Supplementary Figure 10. killer whale (*Orcinus Orca*) – Model metrics, unseen recording-based ROC-Curves and time-wise precision and recall, Network prediction settings: window-length $\varepsilon = 2000$ ms, step-size $\kappa = 500$ ms (see ORCA-SPOT[34]) (created via Inkscape[39], Version 0.92.3)

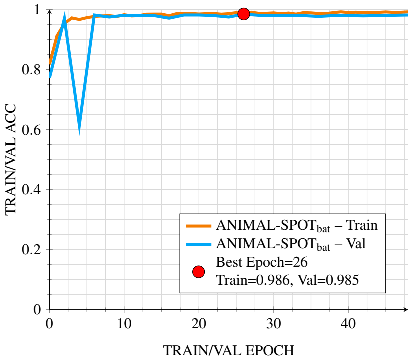

(1.1) Accuracy (ACC)

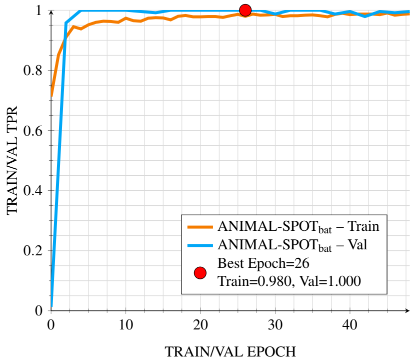

(1.2) True-Positive-Rate (TPR)

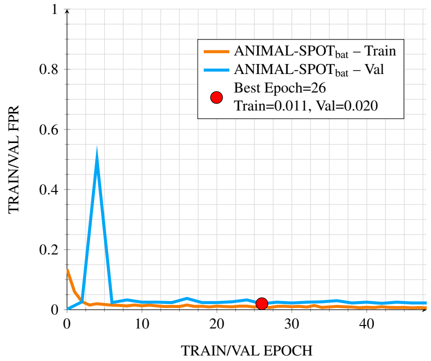

(1.3) False-Positive-Rate (FPR)

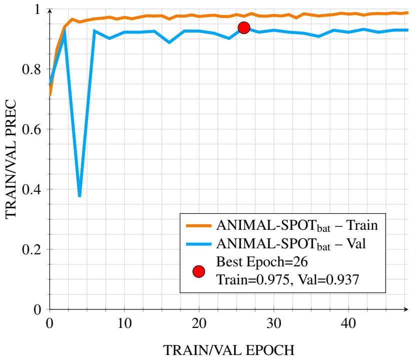

(1.4) Precision (PREC)

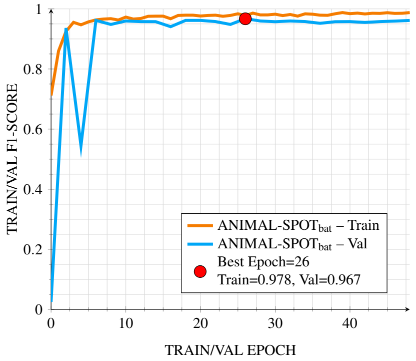

(1.5) F1-Score

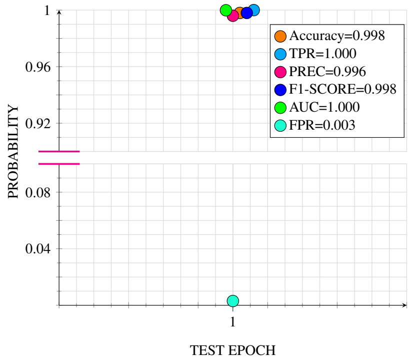

(1.6) Test Metrics

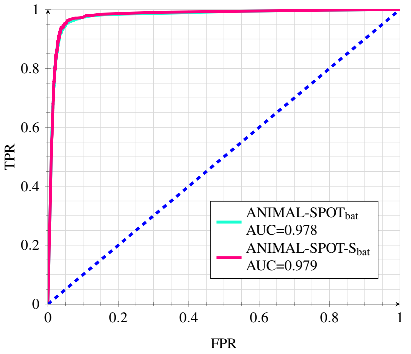

(1.7) ROC-Curve Recording A (high)
Duration [s] = 18.39
Target [s] = 2.47, Noise [s] = 15.92
Predicted Frames = 18,384

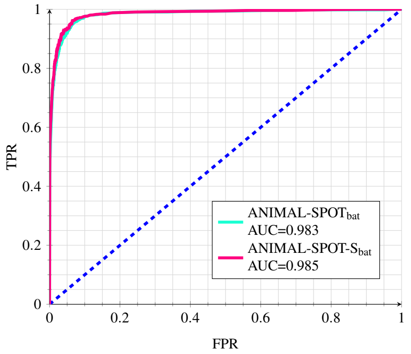

(1.8) ROC-Curve Recording B (med)
Duration [s] = 18.87
Target [s] = 1.50, Noise [s] = 17.37
Predicted Frames = 18,868

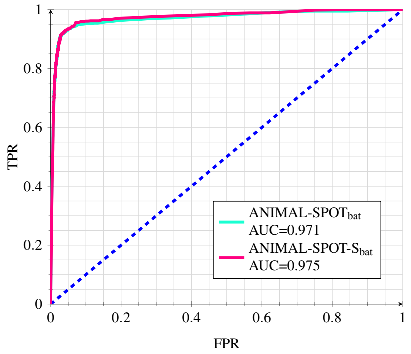

(1.9) ROC-Curve Recording C (low)
Duration [s] = 35.33
Target [s] = 1.75, Noise [s] = 33.58
Predicted Frames = 35,322

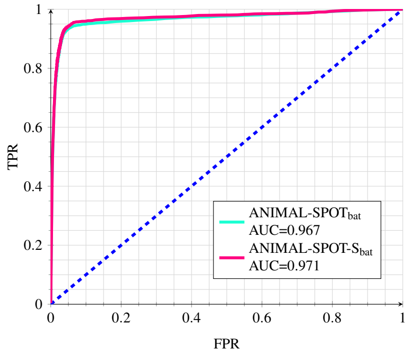

(1.10) Overall ROC-Curve
Duration [s] = 72.59
Target [s] = 5.72, Noise [s] = 66.87
Predicted Frames = 72,574

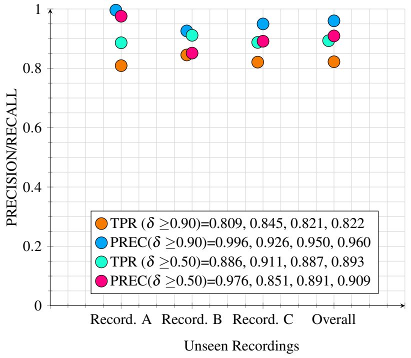

(1.11) ANIMAL-SPOT-S$_{bat}$ – Precision and Recall (TPR)
Threshold ($\delta \geq$0.90 and $\delta \geq$0.50)
Annotation merge factor ξ [s] = 0.0025
Prediction overlap factor λ [s] = 0.0025

Supplementary Figure 11. Pygmy pipistrelle (*Pipistrellus Pygmaeus*) – Model metrics, unseen recording-based ROC-Curves and time-wise precision and recall, Network prediction settings: window-length $\varepsilon = 5$ ms, step-size $\kappa = 1$ ms (created via Inkscape[39], Version 0.92.3)

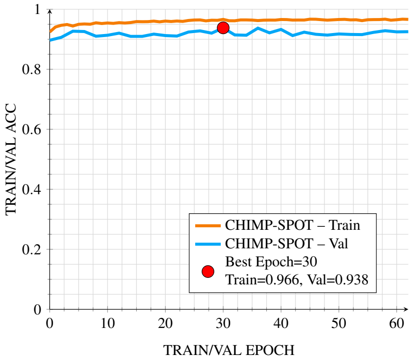

(1.1) Accuracy (ACC)

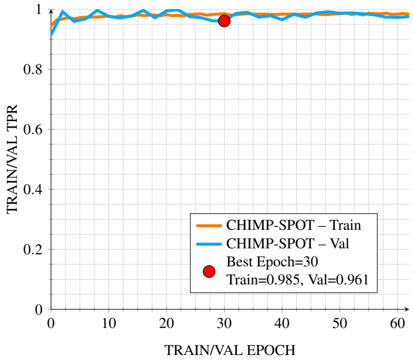

(1.2) True-Positive-Rate (TPR)

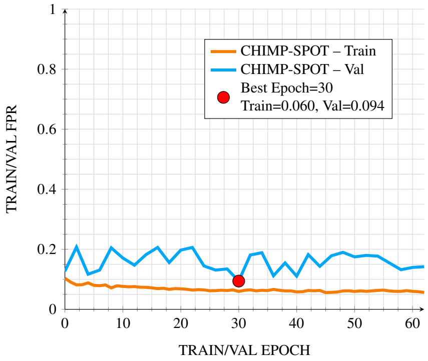

(1.3) False-Positive-Rate (FPR)

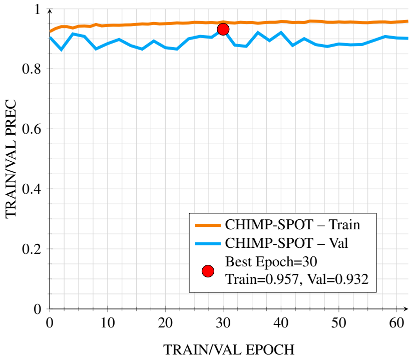

(1.4) Precision (PREC)

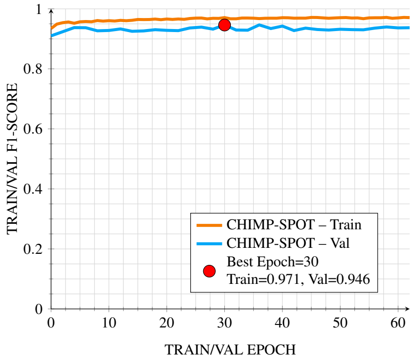

(1.5) F1-Score

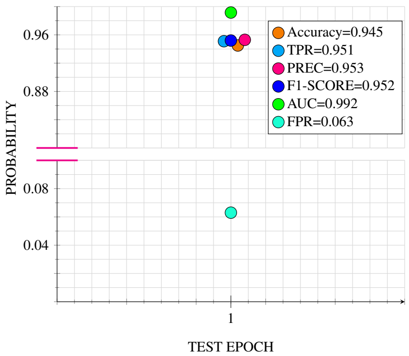

(1.6) Test Metrics

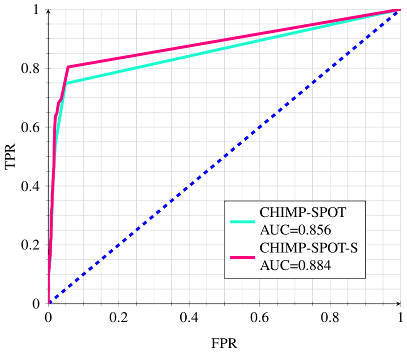

(1.7) ROC-Curve Recording A (high)
Duration [s] = 1,800.00
Target [s] = 79.05, Noise [s] = 1,720.95
Predicted Frames = 17,988

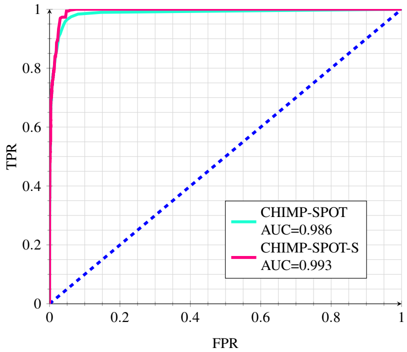

(1.8) ROC-Curve Recording B (med)
Duration [s] = 1,800.00
Target [s] = 75.75, Noise [s] = 1,724.25
Predicted Frames = 17,988

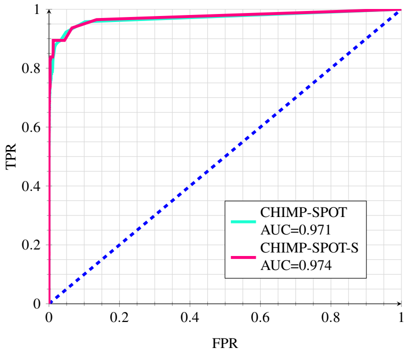

(1.9) ROC-Curve Recording C (low)
Duration [s] = 1,800.00
Target [s] = 18.77, Noise [s] = 1,781.23
Predicted Frames = 17,988

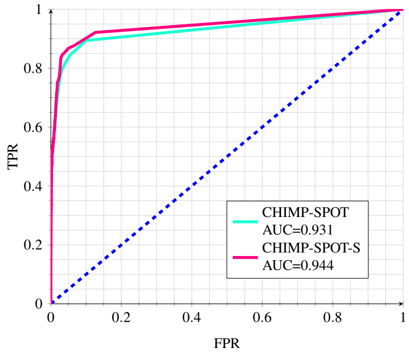

(1.10) Overall ROC-Curve
Duration [s] = 5,400.00
Target [s] = 173.57, Noise [s] = 5,226.43
Predicted Frames = 53,964

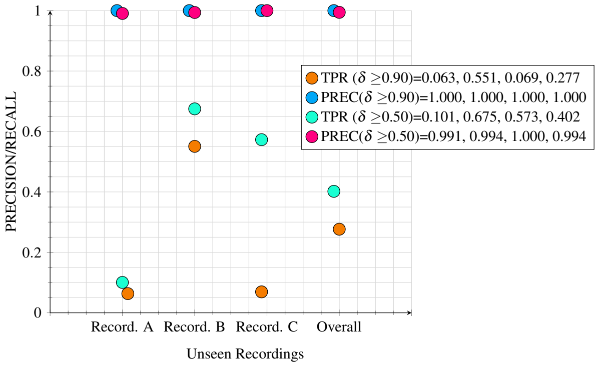

(1.11) ANIMAL-SPOT-S$_{chimp}$ – Precision and Recall (TPR)
Threshold ($\delta \geq 0.90$ and $\delta \geq 0.50$)
Annotation merge factor ξ [s] = 0.65
Prediction overlap factor λ [s] = 0.65

Supplementary Figure 12. chimpanzee (*Pan Troglodytes*) – Model metrics, unseen recording-based ROC-Curves and time-wise precision and recall, Network prediction settings: window-length $\varepsilon = 1300$ ms, step-size $\kappa = 100$ ms (created via Inkscape[39], Version 0.92.3)
